# Sox5 is involved in germ-cell regulation and sex determination in medaka following co-option of nested transposable elements

**DOI:** 10.1186/s12915-018-0485-8

**Published:** 2018-01-29

**Authors:** Manfred Schartl, Susanne Schories, Yuko Wakamatsu, Yusuke Nagao, Hisashi Hashimoto, Chloé Bertin, Brigitte Mourot, Cornelia Schmidt, Dagmar Wilhelm, Lazaro Centanin, Yann Guiguen, Amaury Herpin

**Affiliations:** 1 Physiological Chemistry, Biocenter, University of Würzburg, 97074 Würzburg, Germany; 2 Comprehensive Cancer Center Mainfranken, University Hospital, 97080 Würzburg, Germany; 3 Texas Institute for Advanced Study and Department of Biology, Texas A&M University, College Station, TX 77843 USA; 4 Bioscience and Biotechnology Center, Nagoya University, Furo-cho, Chikusa-ku, Nagoya, Aichi Japan; 5 INRA, UR1037 Fish Physiology and Genomics, F-35000 Rennes, France; 6 Department of Anatomy & Neuroscience, University of Melbourne, Parkville, Victoria 3010 Australia; 7 Centre for Organismal Studies (COS), University of Heidelberg, Heidelberg, Germany

**Keywords:** Exaptation, Master sex-determining gene, Transcriptional rewiring, Medaka, Dmrt1bY, Sox5

## Abstract

**Background:**

Sex determination relies on a hierarchically structured network of genes, and is one of the most plastic processes in evolution. The evolution of sex-determining genes within a network, by neo- or sub-functionalization, also requires the regulatory landscape to be rewired to accommodate these novel gene functions. We previously showed that in medaka fish, the regulatory landscape of the master male-determining gene *dmrt1bY* underwent a profound rearrangement, concomitantly with acquiring a dominant position within the sex-determining network. This rewiring was brought about by the exaptation of a transposable element (TE) called *Izanagi*, which is co-opted to act as a silencer to turn off the *dmrt1bY* gene after it performed its function in sex determination.

**Results:**

We now show that a second TE, *Rex1*, has been incorporated into *Izanagi*. The insertion of *Rex1* brought in a preformed regulatory element for the transcription factor Sox5, which here functions in establishing the temporal and cell-type-specific expression pattern of *dmrt1bY*. Mutant analysis demonstrates the importance of Sox5 in the gonadal development of medaka, and possibly in mice, in a *dmrt1bY*-independent manner. Moreover, Sox5 medaka mutants have complete female-to-male sex reversal.

**Conclusions:**

Our work reveals an unexpected complexity in TE-mediated transcriptional rewiring, with the exaptation of a second TE into a network already rewired by a TE. We also show a dual role for Sox5 during sex determination: first, as an evolutionarily conserved regulator of germ-cell number in medaka, and second, by de novo regulation of *dmrt1* transcriptional activity during primary sex determination due to exaptation of the Rex1 transposable element.

**Electronic supplementary material:**

The online version of this article (10.1186/s12915-018-0485-8) contains supplementary material, which is available to authorized users.

## Background

Sex determination (SD) is one of the most plastic processes in evolution. The trigger for the bipotential undifferentiated embryonic gonad anlage to develop into either testis or ovary can be provided by various signals from the environment, the genetic constitution of the individual, or a mixture of both [1, 2]. Studies of the modes of genetic SD revealed that the genes at the top of the regulatory network and the genes of the network itself are subject to rapid changes in evolution. New master SD genes evolved repeatedly and independently [3]. This situation is particularly obvious in fish, since closely related sister species can have different genetic SD systems or master SD genes [3–7].

Clearly, such a high turnover of genetic determinants can work only if the evolutionary innovations are accompanied by the ability of the respective genes to neo-functionalize or sub-functionalize quickly and efficiently [8, 9]. In addition to changes in protein structure, differences in gene regulation have an important role in evolution and are considered a quick and effective way to adapt gene functions to novelty [10–12]. Hence, the necessity for the transcriptional rewiring of the architecture of the SD regulatory network and connecting novel master SD genes to it requires high-capacity and fast mechanisms. Such a mechanism was proposed by Britten and Davidson almost 50 years ago [13, 14]. They hypothesized that transposable elements (TEs) carry preformed transcription factor binding sites, which, after mobilization and insertion into novel locations of the genome, would contribute novel regulatory features to nearby genes through these motifs. However, examples of authentic co-option, or exaptation [15], of TEs, where most or all gene transcriptions initiate within a TE, remain sparse (see [16] for a review).

Intriguingly, of the handful of examples of this process, one of the best documented comes from a novel SD gene. The master male-determining gene *dmrt1bY* of the teleost fish *Oryzias latipes* (medaka) arose approximately 5 to 10 million years ago from an autosome encompassing the *dmrt1* gene. Dmrt1 is a highly conserved transcription factor that usually functions at a downstream position of the sexual regulatory cascade. In medaka, the *dmrt1* gene was locally duplicated and inserted into another chromosome that became the Y-chromosome [5]. To exert its novel function at its new upstream position, *dmrt1bY* acquired a divergent expression pattern and effector gene profile compared to its autosomal ancestor, *dmrt1a* [5, 17]. We previously showed that this evolutionary innovation, which required a complete rewiring of the regulatory network, was partly brought about by exaptation of a ready-to-use pre-existing *cis*-regulatory element contributed by a TE, called *Izanagi* [17]. This element acts as a silencer. It recruits proteins Dmrt1bY and Dmrt1a to turn off the *dmrt1bY* gene after it has fulfilled its function as the primary male SD gene.

We report here that TE-mediated transcriptional rewiring can reach an unexpected level of complexity that exceeds this simple feedback regulation. We find that another TE, *Rex1*, has jumped into *Izanagi*. Through the disruption of *Izanagi*, *Rex1* immobilized this TE and fixed the Dmrt1-mediated downregulation. Moreover, *Rex1* brought in a preformed regulatory element for the transcription factor Sox5. We show that medaka Sox5 binds to the *sox5*-responsive elements of the *dmrt1bY* promoter and downregulates its transcriptional activity. Interestingly, in vivo analysis of double transgenic fluorescent reporter fish additionally revealed a complementary pattern of expression of both genes. The higher expression of *sox5* correlates with a lower expression of *dmrt1bY* and vice versa. Our results underpin the importance of the *Rex1* TE for the establishment of a new SD mechanism in medaka and likely contribute in establishing the temporal and cell-type specific expression pattern of *dmrt1bY*.

Several transcription factors of the Sox family (Sox3, SRY, Sox 9, and sox8) play crucial roles in SD, but Sox5 has not been previously implicated in SD in any metazoan so far (neither Sox5 in vertebrates, nor its *Drosophila* homologue Sox102F). Interestingly, in medaka, disruption of *sox5* leads to XX female-to-male sex reversal. From an analysis of mutants, we find the critical involvement in gonadal development in medaka by regulating primordial germ cells (PGCs). In overexpression experiments, there is an ectopic induction of germ-cell markers including *dmrt1*. With all necessary notes of caution, our preliminary expression pattern data, also detecting SOX5 expression in the fetal gonad of mice, may indicate an evolutionarily conserved role for SOX5 during early mammalian gonad development.

Our work reveals a dual role for *sox5* during SD: (i) first being an evolutionarily conserved important regulator of germ-cell number in medaka and possibly beyond and (ii) second, de novo regulation of medaka *dmrt1* transcriptional activity during primary SD after it has been recruited for transcriptional rewiring of the *dmrt1* promoter due to exaptation of a TE.

## Results

### Identification of putative Sox5 transcription factor binding sites in the *dmrt1bY* promoter

In an initial analysis of the promoter of the medaka male-determining gene *dmrt1bY*, we found that after duplication from its autosomal progenitor *dmrt1a*, an insertion of an *Izanagi* DNA transposon brought in a novel transcriptional repressor element [17]. It functions by binding Dmrt1a and Dmrt1bY transcription factors and is essential for the downregulation of *dmrt1bY* after fulfilling its SD function in the male gonad.

**Fig. 1:**
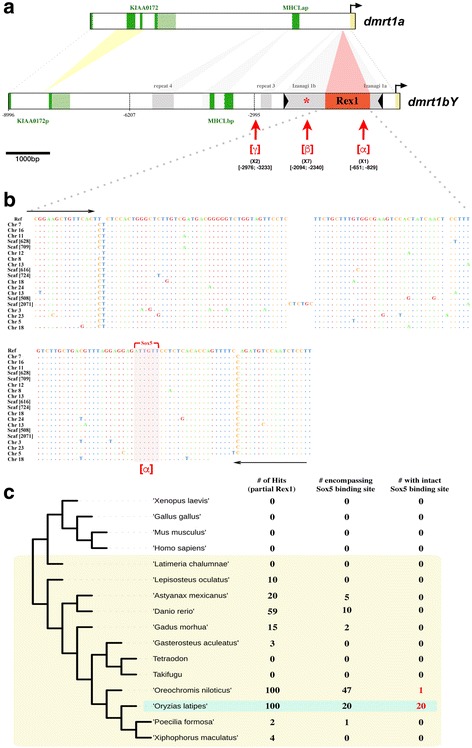
Comparative analysis of the *dmrt1a* and *dmrt1bY* co-ortholog promoters and presence of *Rex1* element sequences in the genomes of selected fish species. **a** Comparative analysis of the medaka *dmrt1* co-ortholog promoter regions. Differences in length for the two promoter regions are caused by *Rex1* and *Izanagi* transposable elements as well as repeats 3 and 4 that were inserted into the *dmrt1bY* promoter after the duplication event that gave rise to the *dmrt1bY* gene approximately 10 million years ago [17]. Regions α, β, or γ (brackets [] underlined in red) contain multiple Sox5 binding sites within *Rex1*, *Izanagi*, and repeat 3, respectively, that have been subjected to chromatin immunoprecipitation (ChIP) (see also Additional file 1: Figure S1). The red star (*) identifies the Dmrt1 binding site described in [17]. **b** Alignment of the Y-chromosomal *Rex1* element together with the 19 remaining *Rex1* copies encompassing the sox5 binding site in the medaka genome. Dots indicate conserved nucleotides. Black arrows define primers used for chromatin immunoprecipitation. **c** Presence of *Rex1* element (i) partial sequences, (ii) sequences encompassing the dmrt1bY-nested sox5 binding site, and (iii) sequences encompassing the dmrt1bY-nested sox5 binding site with the intact sox5 binding site in the genomes of medaka (*Oryzias latipes*), tilapia (*Oreochromis niloticus*), zebrafish (*Danio rerio*), cavefish (*Astyanax mexicanus*), cod (*Gadus morhua*), gar (*Lepisosteus oculatus*), stickleback (*Gasterosteus aculeatus*), platy (*Xiphophorus maculatus*), Amazon molly (*Poecilia formosa*), fugu, tetraodon, and coelacanth (*Latimeria chalumnae*)

In addition to this repressor element [17], the promoter region contains an unexpectedly high density of putative binding sites for Sox5 (see the [*β*] region in Fig. 1a and [17]). It harbors seven Sox5 binding sites; a random prediction would expect only 0.46 sites over the whole sequence. Interestingly, a unique putative Sox5 binding site is also found within a *Rex1* TE [*α*], and two within repeat 3 [*γ*] of the *dmrt1bY* proximal promoter region (see [*α*], [*β*], or [*γ*] in Fig. 1a and Additional file 1: Figure S1 for sox5 binding site locations). These regions were all inserted into the promoter after the duplication event and, thus, during the evolution of the novel male-determining function of *dmrt1bY*.

In particular, the Sox5 binding site nested within the *Rex1* TE of the *dmrt1bY* proximal active promoter showed high prediction probability (weight 9.4, *p* value 5.4 × 10^-5^, lnPval -9;831; [*α*] in Fig. 1a, see also “Methods” for the positional weight matrix employed). To address the question whether this Sox5 binding site has evolved de novo after insertion or was already an integral part of the *Rex1* element that was inserted into the *dmrt1bY* promoter, we blasted the *Rex1* sequence of the *dmrt1bY* promoter against the medaka and other fish genomes (Fig. 1b,c). *Rex1* elements are present in 100 copies in the genome of medaka. Many copies are also found in tilapia (100) and zebrafish (59), but there are fewer in cavefish (20), cod (15) and gar (10). They are scarce in stickleback (3), platyfish (4), and Amazon molly (2), and absent in fugu, tetraodon, and coelacanth (Fig. 1c). Interestingly, among Rex*1* elements, the region encompassing the predicted *sox5*-binding site is very poorly conserved despite being part of the reverse transcriptase-coding region (region 6) of the *Rex1* element (Additional file 2: Figure S2). It can be detected with some divergence to the consensus sequence in only 47, 20, 10, 5, 2, and 1 copies in tilapia, medaka, zebrafish, cavefish, cod, and Amazon molly, respectively (Fig. 1c). An intact sox5 binding site is predicted in only two species. In tilapia, a single copy has a high-fidelity site, whereas 20 copies in medaka, including the one in the *dmrt1bY* promoter, have putatively intact *sox5* binding sites (Fig. 1b,c and Additional file 3: Table S1). Hence, *Rex1*-nested *sox5* binding sites appear to be a medaka-specific feature.

Sox5 has been correlated with *dmrt1* expression in zebrafish [18] and the wrasse, *Halichoeres tenuispinis* [19], in in vitro promoter studies. Thus, we hypothesized that the identified *sox5* binding sites could be involved in the transcriptional rewiring of *dmrt1bY*.

### Sox5 binds to the putative Sox5-responsive elements of the dmrt1bY promoter with different affinities

**Fig. 2:**
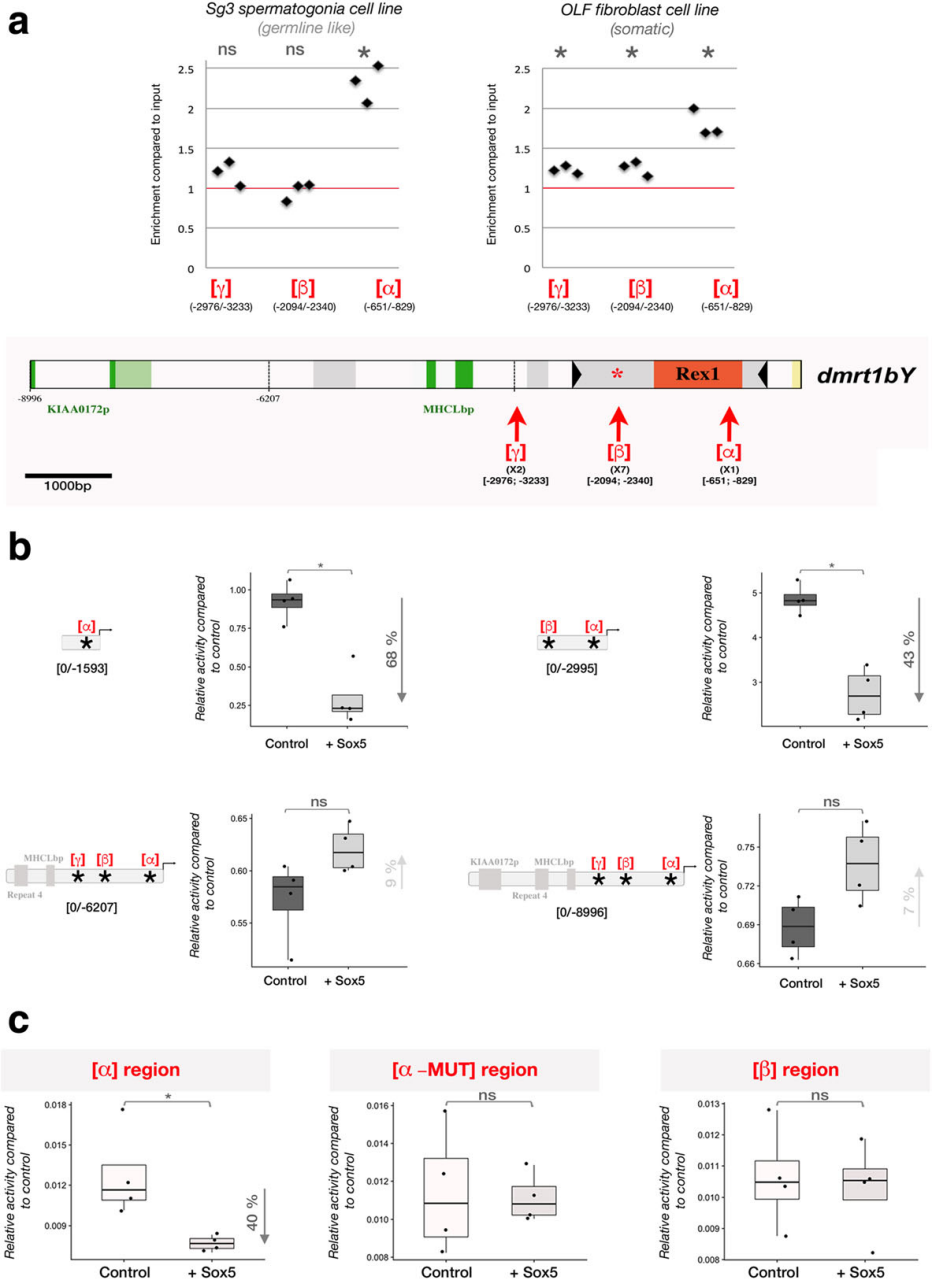
Analysis of Sox5 binding to the *dmrt1bY* promoter and regulation of *dmrt1bY* promoter activity upon modulation of Sox5 expression. **a** Chromatin immunoprecipitation (ChIP) of Sox5 binding to regions α, β, or γ of the *dmrt1bY* proximal promoter. Transient transfection of a flagged and tagged version of Sox5 into either medaka spermatogonial or fibroblast cell lines and subsequent immunoprecipitation (FLAG antibody) followed by the quantitative real-time polymerase chain reaction. Results are presented as enrichment compared to the input and correspond to three independent immunoprecipitations for each region (α, β, or γ). Statistical significance was assessed with the Wilcoxon–Mann–Whitney test (*N* = 3). **b1–b4** Analysis of *dmrt1bY* proximal promoter activity after Sox5 transient transfection into the medaka spermatogonial cell line (Sg3). Deletions of the 5′ *dmrt1bY* promoter were generated: **b1** α region, **b2** α and β regions, **b3, b4** α, β, and γ regions. Transcriptional activity was quantified in the absence (control, -Sox5) or presence (+Sox5) of Sox5. Statistical significance was assessed with the Wilcoxon–Mann–Whitney test (*N* = 4). **c** Detailed analysis of the transcriptional activity of the alpha (α), alpha-mutant (α-MUT), and beta (β) fragments. Statistical significance was assessed with the Wilcoxon–Mann–Whitney test (*N* = 4). * *p* value ≤ 0.05, ** *p* value ≤ 0.01. ns non-significant, OLF *Oryzias latipes* fibroblast, Sg3 *Oryzias latipes* spermatogonial cell

To assess the relevance of the predicted Sox5 binding sites [*α*], [*β*], and [*γ*] (see Fig. 1a,b) in the *dmrt1bY* promoter, two different medaka cell lines, *Oryzias latipes* spermatogonial (Sg3) and fibroblast (OLF) cells, were transfected with a FLAG-tagged version of Sox5 and then subjected to chromatin immunoprecipitation (ChIP) using an anti-FLAG antibody. DNA amplification with specific sets of primers from the Sox5 immunoprecipitates revealed that the Sox5 protein binds to the predicted sites ([*α*], [*β*], and [*γ*]) of the *dmrt1bY* promoter. However, much stronger binding is apparent for the proximal site [*α*] (up to tenfold higher enrichment, Fig. 2a) located within the *Rex1* element. Of note, although two DNA-binding sequences for Sox5 were predicted within the *dmrt1a* proximal promoter (at positions [-286/-300] and [-1100/-1116] upstream of the ATG), they do not appear to be functional, as Sox5 does not target them in the ChIP experiments (data not shown).

Of note, the predicted *sox5* binding region [*β*] has previously been shown to overlap with a high affinity binding site for Dmrt1 [17]. Hence, competition between Sox5 and Dmrt1 for access to this sequence motif cannot be excluded. If Dmrt1 already occupies the motif, this might explain the low amount of recovery in the Sox5 ChIP experiment.

### Sox5 downregulates the activity of the dmrt1bY promoter

Next, we examined (i) the respective contributions of each part of the promoter for *dmrt1bY* transcriptional regulation and (ii) the direction of that regulation (up- or downregulation) using transcriptional reporter assays. Thus, 5′ deletions of the *dmrt1bY* promoter ([0/-1593], [0/-2995], [0/-6207], and [0/-8996]) were produced and analyzed after transient transfection for their ability to drive luciferase expression (Fig. 2b).

In the medaka spermatogonial cell line, the highest promoter activity was detectable for the [0/-2995] proximal promoter region encompassing sites [*α*] and [*β*] (Fig. 2b2). Promoter activity was significantly lower (Fig. 2b3,4), when more distal sequences ([-2996/-6207]) containing site [*γ*] were present in the construct. The shortest proximal promoter region (encompassing site [*α*] only) had intermediate transcriptional activity (Fig. 2b1). Interestingly, the transcriptional activity of the most proximal parts of the *dmrt1bY* promoter—encompassing the sites [*α*] in *Rex1* (Fig. 2b1) and [*β*] in *Izanagi* (Fig. 2b2)—was reduced by between 43% and 68% when Sox5 was overexpressed (Fig. 2b1,2). This effect of Sox5 overexpression was not apparent for the longer constructs including further distal sequences displaying strong constitutive repression (Fig. 2b3,4). Of note, the highest repression of *dmrt1bY* promoter transcriptional activity was observed for the proximal promoter region encompassing the [*α*] site in *Rex1* (68% in Fig. 2b1). Next, modulation of transcriptional activity was tested for the [*α*], [*α*]-MUT, and [*β*] regions alone (Fig. 2c). Interestingly, only the [*α*] region was able to downregulate promoter activity (by about 40%) when fused to a minimal thymidine kinase promoter. Neither the [*β*] or a mutated version of the [*α*] region ([*α*]-MUT) were able to modulate the activity of the minimal thymidine kinase promoter (Fig. 2c).

**Fig. 3:**
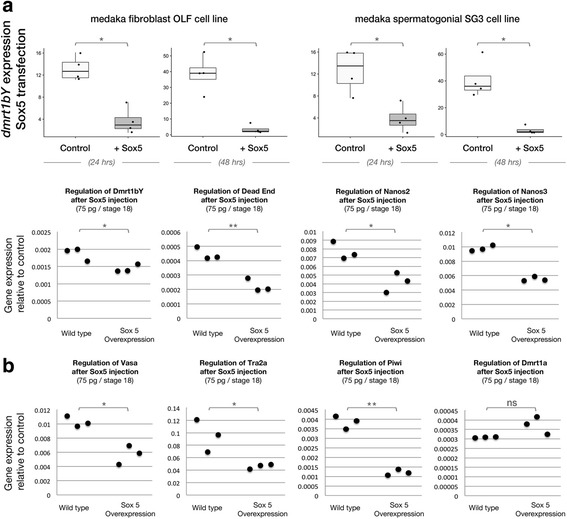
Effects of Sox5 modulation on *dmrt1bY* gene expression. Analysis of *dmrtbY* **a** transcriptional regulation after *sox5* transient transfection in either spermatogonial or fibroblast medaka cell lines at 24 or 48 h post-transfection. Dataset results of four independent transfections. Statistical significance was assessed with the Wilcoxon–Mann–Whitney test (*N* = 4; * *p* value ≤ 0.05, ** *p* value ≤ 0.01). **b** Overexpression of sox5 was stimulated in fish eggs. The expression of *dmrt1bY* and germ-cell markers (*nanos2*, *nanos3*, *dead-end*, *vasa*, *tra2a*, and *piwi*) were monitored at stage 18 of development and compared to wild-type fish embryos. Dataset results of three independent transfections. Statistical significance was assessed with the *t*-test (*N* = 3 and each replicate is a pool of 25 eggs; * *p* value ≤ 0.05, ** *p* value ≤ 0.01). ns non-significant, OLF *Oryzias latipes* fibroblast, Sg3 *Oryzias latipes* spermatogonial cell

To obtain a more precise readout for the regulation of medaka *dmrt1bY* expression by Sox5, spermatogonial and fibroblast medaka cell lines were transiently transfected with a Sox5-expressing construct and endogenous *dmrt1bY* expression was quantified by the real-time polymerase chain reaction (RT-PCR) at different time points post-transfection (24, 48, and 72 h). Our findings reveal that the transcription of *dmrt1bY* is highly repressed in the presence of Sox5 (up to 90% 48 h after transfection, Fig. 3a) in both the OLF and Sg3 medaka cell lines.

Next, to validate our in vitro results, Sox5 coding mRNAs were microinjected into one-cell-stage embryos, and the expression of *dmrt1bY* and several germ-cell markers (*nanos2*, *nanos3*, *dead-end*, *vasa*, *tra2a*, and *piwi*) was monitored (Fig. 3b). The results confirm the transient cell transfection experiments and demonstrated that in vivo Sox5 can act as a negative modulator of *dmrt1bY* expression (Fig. 3b). Overexpression of Sox5 also resulted in the repression of all analyzed germ-cell genes regardless of their intrinsic endogenous expression levels (Fig. 3b). Our in vivo results identify Sox5 as a strong negative regulator of germ-cell gene expression, including *dmrt1bY*. Interestingly, although most of the germ-cell marker genes (*nanos3*, *dead-end*, *vasa*, and *piwi*) are maternally deposited, their lower relative abundances compared to controls after Sox5 overexpression are likely attributable to a total arrest of background transcription after zygotic transcription started, or possibly accelerated mRNA decay.

### Expression of sox5 during early gonad primordium formation

**Fig. 4:**
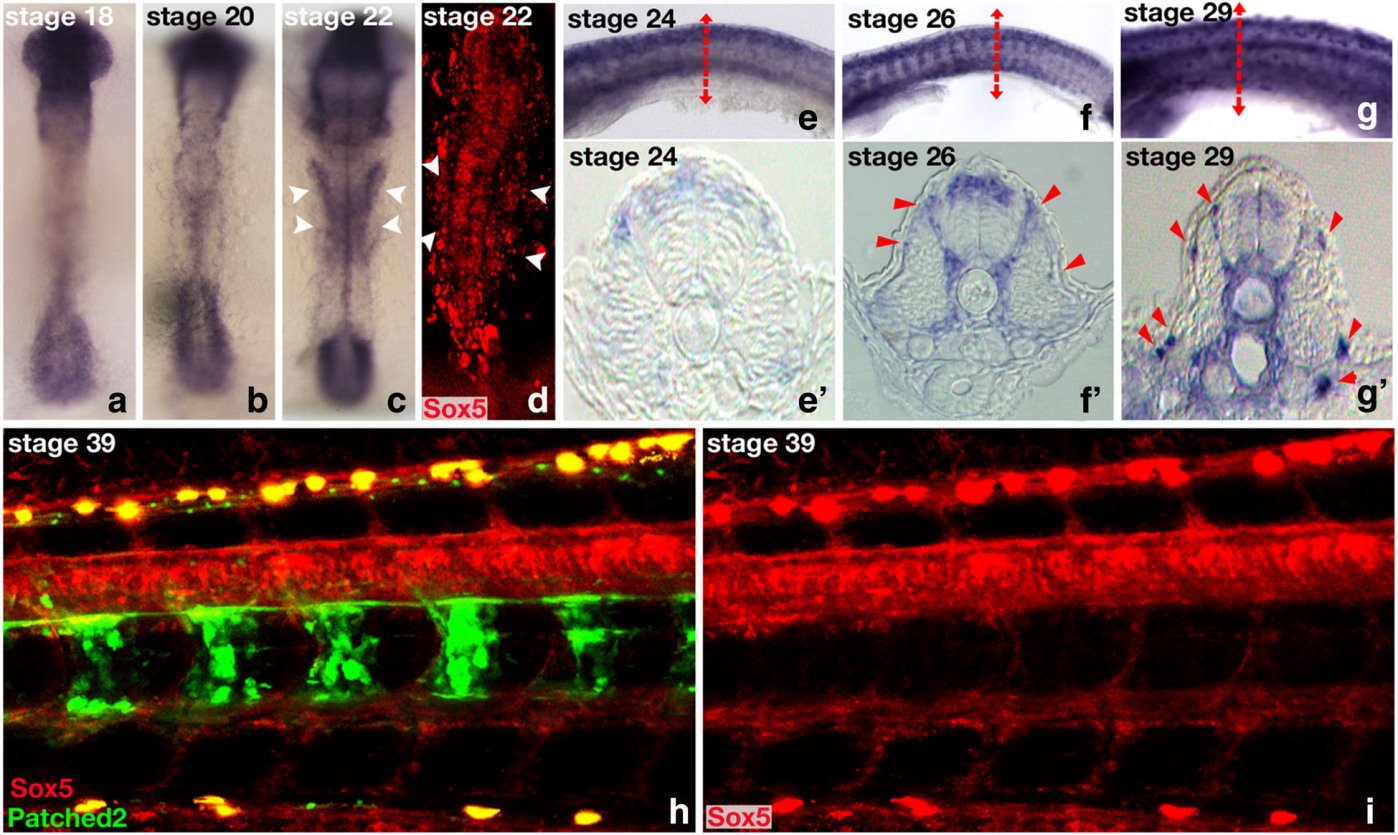
Expression of medaka *sox5* during embryogenesis. **a** to **c** and **e** to **g** Medaka *sox5* expression investigated by whole-mount in situ hybridization or **d**, **h**, and **i** fluorescence using a transgenic reporter line for which a 3288-bp *sox5* promoter fragment drives the expression of mCherry. **a-c** Between stages 18 and 22, *sox5* mRNA localizes predominantly in the head and tail bud regions of the embryos. **c,d** At stage 22, additional expression is detected in the lateral plate mesoderm of the embryos (arrows). **e**–**g'** From stage 24 onward, sox5 expression spans over the dorsal neural tube and pre-migratory neural crest cells (arrowheads). **g,g'** At stage 29, *sox5* expression is also seen in ventral migrating neural crest cells (arrowheads). **h** and **i** Fluorescent *sox5* expression is monitored in the neural tube and neural crest cells of hatching embryos (stages 38/39). **h** For comparison, *patched2* highlights the notochord at stage 39 [11]

**Fig. 5:**
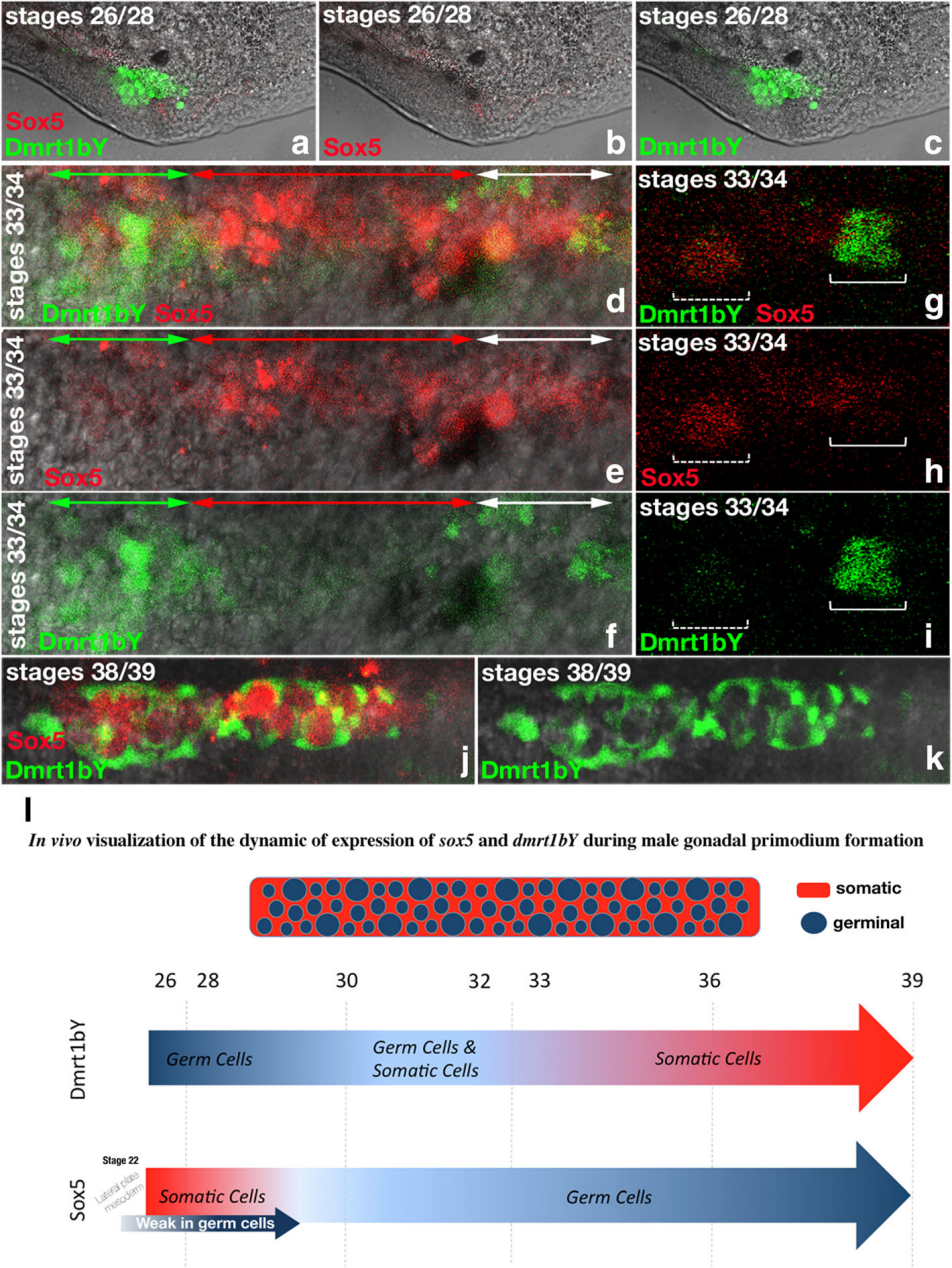
Comparative analysis of *sox5* and *dmrt1bY* expression dynamics during gonadal primordium formation. Expression of *sox5* compared to *dmrt1by* in a double transgenic fluorescent reporter line. **a**–**c** During early gonadal formation, *sox5* is first detected in the somatic tissues surrounding the germ cells at stages 26 to 28. At the same time, *dmrt1bY* is expressed in germ cells. **d**–**i** By stages 33 to 34, *sox5* expression becomes restricted to the germ cells. *dmrt1bY* is also expressed in the germ cells at those specific stages of development. Variations within the respective levels of sox5 and dmrt1bY expression are clearly observable (**d** compared to **e** and **f** and **g** compared to **h** and **i**). **j**,**k** Around hatching (stages 38/39), the expression of *sox5* strengthens in all germ cells while parallel *dmrt1bY* expression quickly switches from germ cells only to somatic germ-cell-surrounding cells only. **l** In vivo visualization of the dynamics of expression localization of *sox5* and *dmrt1bY* during male gonadal primordium development. The expression of *sox5* and *dmrt1bY* is highly dynamic during primordium gonadal formation, switching from somatic to germ cells and vice versa, respectively, from stage 26 until hatching. Being mutual repressors of each other, a seesaw of expression is observed, finally finely restricting *dmrt1bY* expression in the somatic part of the primordium gonad. Blue and red represent cellular expression localizations only and should not been interpreted as expression levels

Medaka *sox5* mRNA, which is expressed in embryonic and early larval development, has a distinct spatially and temporarily restricted expression pattern (Fig. 4). Between stages 18 and 22, *sox5* transcripts localize mainly in the head and tail bud regions of the embryos (Fig. 4a–d). At stage 22, expression is additionally detected in the lateral plate mesoderm (arrowheads in Fig. 4c,d), from which the somatic gonadal primordium will develop shortly thereafter [20]. Later, *sox5* expression is observed over the entire dorsal neural tube (Fig. 4e,e',f,f') and pre-migratory neural crest cells (NCCs, Fig. 4f'). At stage 29, *sox5* transcripts are present in migrating NCCs ventrally [21] (Fig. 4g and arrowheads in g'). A higher resolution of the dynamic expression pattern of *sox5* in vivo was obtained with a transgenic line, which has the 3288-bp upstream promoter region of *sox5* fused to a fluorescent (*mCherry*) reporter. The reliability of *sox5* gene expression was confirmed by comparison of the observed fluorescence with the in situ hybridization data (Fig. 4c and f' compared to d, h, and i, respectively). *Sox5* promoter-driven fluorescence was detected as early as stage 22 in the lateral plate mesoderm (Fig. 4d). This region has been shown to have already the properties of a gonadal field because somatic gonadal precursors arise from the most posterior part of the lateral plate mesoderm [20]. Consistently, at stages 26/28, when the gonadal primordium just has formed, faint *sox5* expression is still observable in the somatic tissues surrounding the germ cells that express Dmrt1bY (Fig. 5a–c).

At stages 33 to 34, the gonadal expression of *sox5* is restricted to the germ cells (Fig. 5d–i). Of note, variations in the levels *of sox5* expression are clearly visible between different germ cells (Fig. 5d,e). Interestingly, *dmrt1bY* (Dmrt1bY:GFP) is also expressed in germ cells at that specific stage of development [22] and displays variations in expression between individual germ cells [23, 24] (Fig. 5d,f). Analysis of the [Sox5:mCherry and Dmrt1bY:GFP] double transgenic line revealed a complementary pattern of expression of both genes: those germ cells that have a higher expression of *sox5* have a lower expression of *dmrt1bY* and vice versa (Fig. 5d–i).

During the following developmental stages (stages 38/39), the expression of *sox5*:*mCherry* increases in all germ cells, whereas *dmrt1bY:GFP* concurrently switches from germ cells to the somatic, germ-cell-surrounding cells (Fig. 5j,k). Taken together, these results show that expression of *sox5* and *dmrt1bY* is highly dynamic during gonadal primordium formation, switching from germ cells to somatic cells (Fig. 5l).

To determine whether the expression of sox5 during early gonadal development is a medaka-specific feature or is more widely conserved, we used immunofluorescence on 13.5 and 14.5 days post coitum (dpc) mouse embryos. This revealed that SOX5 was expressed in peritubular myoid cells surrounding cords in the fetal testis (Additional file 4: Figure S3, left and middle panels) and in a subset of somatic and germ cells in the fetal ovary (Additional file 4: Figure S3, right panels). Of note, a substantial fraction of SOX5 expression also displayed cytoplasmic localization. Although reported for other SOX factors (see SOX2 [25], SOX9 [26, 27], and [28] for reviews), the functional significance of the SOX5 non-exclusive nuclear localization in mice gonads remains to be investigated.

### Expression of sox5 in adult gonads

**Fig. 6:**
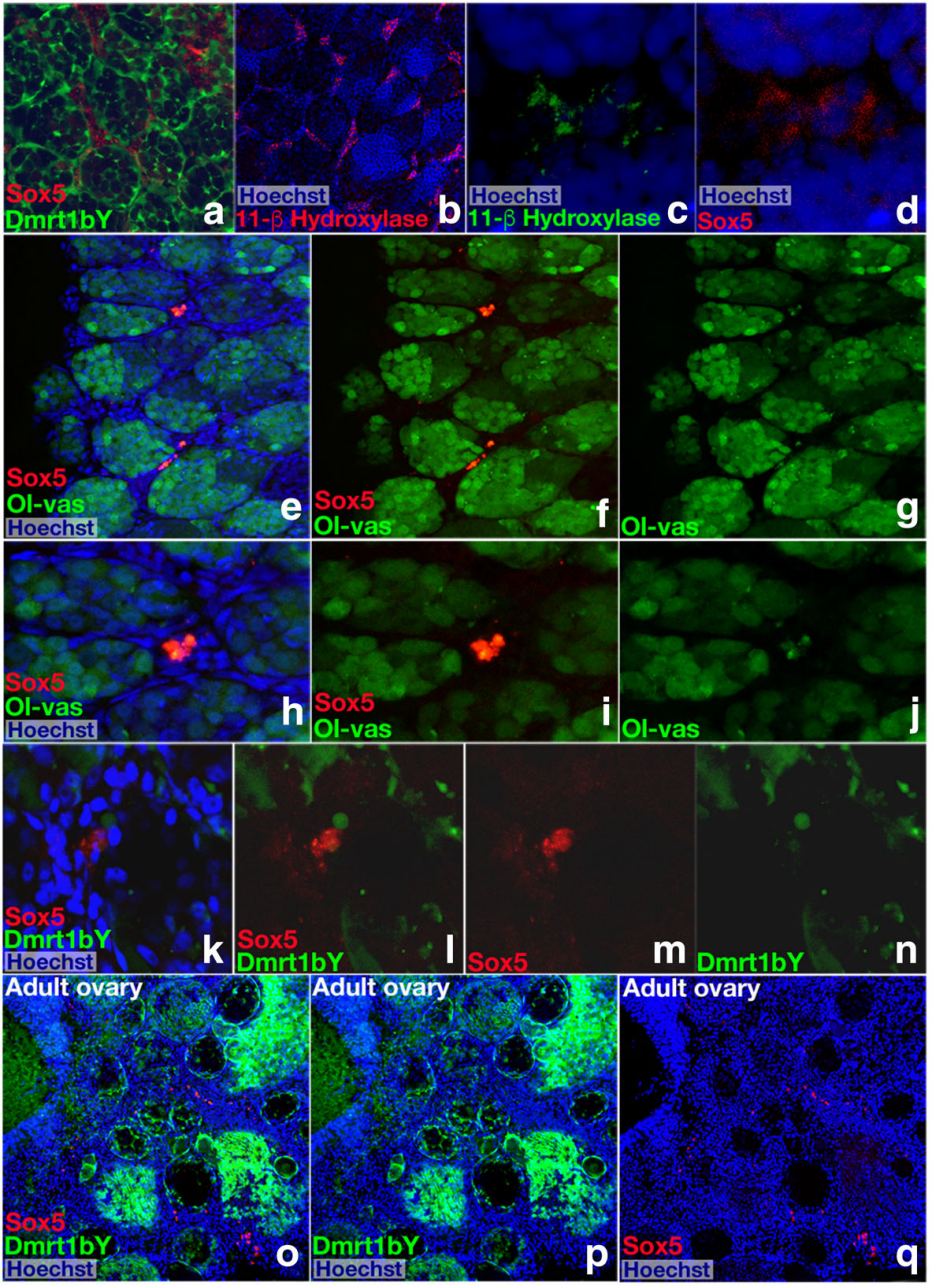
Expression of *sox5* in adult gonads. **a**–**d** In adult testes, sox5 fluorescence is restricted to the cells located between the lobules where the germ and Sertoli cells lie. **a**,**b** In double transgenic reporter fish, fluorescence of sox5 and dmrt1bY (marking the Sertoli cells) is distinct. **c, d** The interlobular expression of *sox5* co-localizes with 11-β-hydroxylase, a marker of Leydig cells. **e**–**j** Expression of sox5 is also detected in another discrete population of cells between but always close to the germinal lobules. **h**-**j** Small in size, these *sox5*-positive cells also express *vasa*, a specific marker of germ cells. **k**–**n** The sox5- and vasa-positive cells do not express *dmrt1bY*. **o**–**q** In adult ovaries, only extremely few sox5 positive cells are detected.

Given reduced *dmrt1bY* expression in the fully developed testis [29], we also monitored the expression of *sox5* in fully mature gonads of both sexes (Fig. 6). In adult testes, *sox5* fluorescence was mainly restricted to the interlobular cells (see Fig. 6a–d). In contrast, *dmrt1bY* expression is clearly localized within the Sertoli cells [17] of the testicular lobules (Fig. 6a). No co-localization of *dmrt1bY* and *sox5* transcripts whatsoever was observed (Fig. 6a). To define the nature of the interlobular *sox5*-positive cells better, immunofluorescence of 11-β-hydroxylase protein, a specific marker of Leydig cells, was performed (Fig. 6b). Interstitial cells of Leydig are found adjacent to the seminiferous lobules in the testes and produce androgens [30]. A perfect co-localization of *sox5* transcripts and 11-β-hydroxylase protein confirmed that the interlobular *sox5*-positive cells are indeed Leydig cells (Fig. 6c,d). Interestingly, another discrete population of *sox5*-positive cells is discernable between but close to the lobules (Fig. 6e–g). These cells are very small compared to their neighboring germ cells, and their nuclei appear compact (Fig. 6h–j). The expression of the germ-cell marker *vasa* (Fig. 6e–j) assigns these cells to the germ-cell lineage, which are probably at a very early stage of differentiation. These germ cells do not express *dmrt1bY* (Fig. 6k–n). The adult ovary displays only a very few s*ox5*-positive cells with small and condensed nuclei (Fig. 6o–q). At present, the identity of these cells is difficult to ascertain, but oocytes and somatic supporting cells (granulosa or theca cells) can be excluded from their morphology and location.

### PGC number is decreased in sox5 mutants

**Fig. 7:**
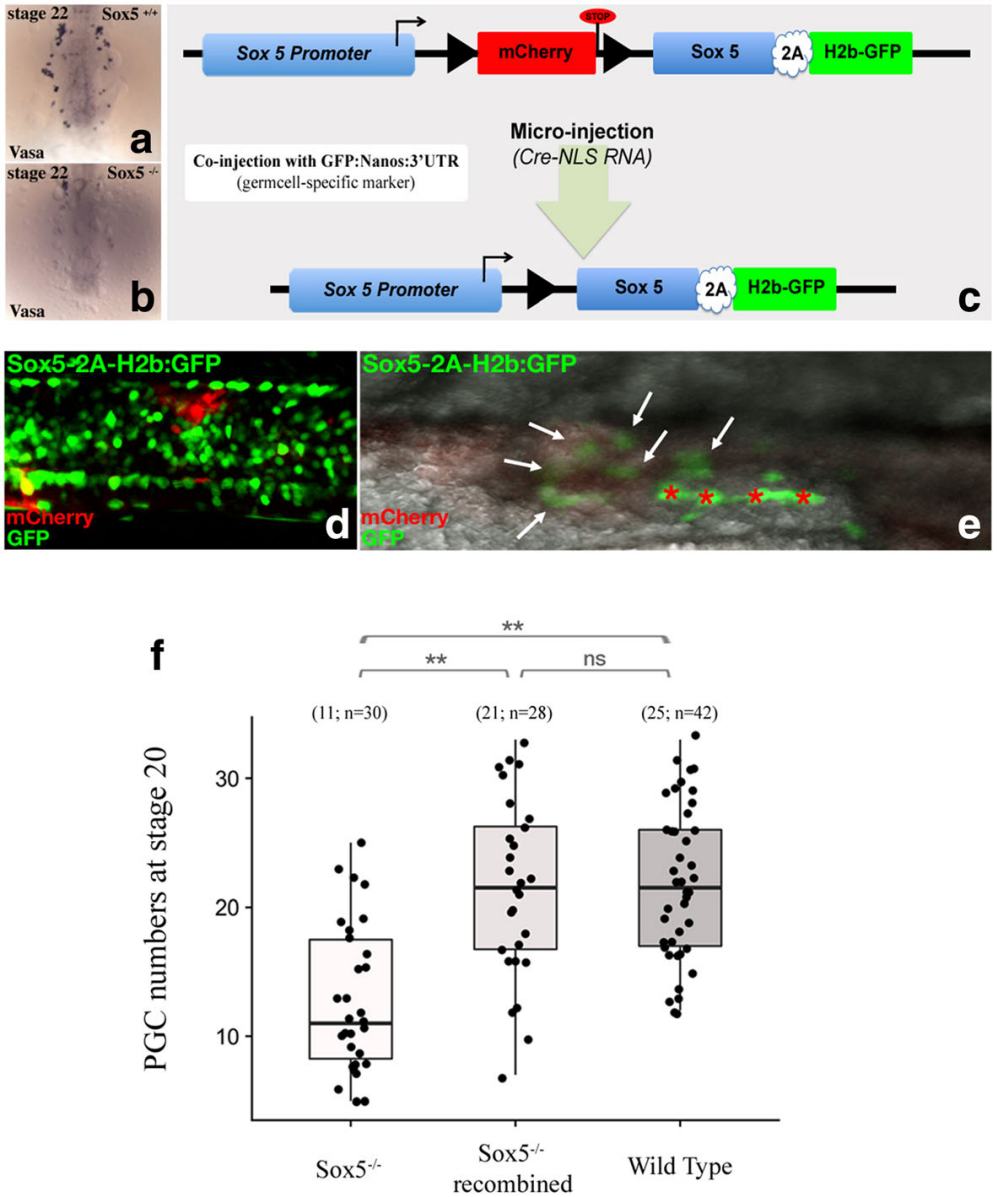
Regulation of PGC numbers by Sox5. **a**,**b** As early as stage 22, a drastic reduction of the germ-cell number (*vasa* in situ hybridization) is observed in sox5^-/-^ mutants compared to wild-type embryos. **c** For conditional knock-in and rescue of the *sox5*^*-/-*^ mutant fish, a transgenic line expressing *sox5* was produced. See “Methods” for details. **d**,**e** In vivo visualization of the effective recombination and expression of *sox5* is apparent after a switch from red cytoplasmic to green nuclear-localized fluorescence. After *Cre recombinase* injection at the one-cell stage, an almost total recombination is observed, leading to the expression of the *sox5* transcript as monitored by green fluorescence. Stars indicate auto-fluorescent pigment cells and arrows indicate recombined germ cells. **f** Germ-cell numbers in *sox5* mutant medaka after in vivo recombination and expression of *sox5*. Of note, for that specific experiment, embryos were additionally injected with a GFP-Nanos 3′ untranslated region (UTR) mRNA construct allowing effective PGC monitoring. Statistical significance was assessed with the Wilcoxon–Mann–Whitney test (*N* = 30, 28, and 42 for Sox5^-/-^, Sox5^-/-^ recombined, and wild-type embryos, respectively; * *p* value ≤ 0.05, ** *p* value ≤ 0.01). GFP green fluorescent protein, ns non-significant, PGC primordial germ cell

To obtain functional data on the role of *sox5* in gonadal development, we next analyzed early gonadal development in mutants. The *ml-3* mutant (N541S) is a naturally occurring mutation, for which a premature stop codon results in the production of a truncated Sox5 protein lacking the HMG box domain and causing Sox5 loss of function [21, 31, 32]. Considering that *sox5* is first expressed in the lateral plate mesoderm and then in germ cells during primordial gonad formation, we investigated whether Sox5 plays a role in regulating PGC number. Whole-mount in situ hybridizations utilizing the PGC-specific marker *vasa* were performed in wild-type and *sox5*^*-/-*^ mutant fish embryos (Fig. 7a,b). At stage 22, during the formation of the primordial gonad when *sox5* has a first expression peak in wild-type embryos, a drastic reduction in the PGC number is evident in mutants (Fig. 7a compared to b). This emphasizes a possible role for Sox5 as being a regulator of PGC proliferation although such a reduction in PGC numbers might also be ascribed to reduced proliferation, reduced survival, or defects in fate specification.

### PGC number is rescued in sox5 mutants by in vivo conditional knock-in of Sox5

Given that absence of functional Sox5 expression in the *sox5*^-/-^ mutant resulted in a reduced number of PGCs, we next attempted to rescue the gonadal phenotype by wild-type *sox5* expression to show that this gene is crucial in regulating PGC numbers. Thus, we established a transgenic line that expresses *sox5* after controlled homologous recombination in the *sox5* mutant genetic background (Fig. 7c). In this line, the *sox5* promoter drives the expression of a mCherry-stop cassette flanked by *LoxP* sites. This cassette is followed by the wild-type *sox5* open reading frame (ORF) fused to a 2A self-cleaving system [33] with nuclear-addressed green fluorescent protein (GFP) (H2B-GFP) (Fig. 7c). After injection of mRNAs encoding for the *Cre-NLS* protein and effective recombination, the mCherry-stop cassette is excised, and the *sox5* ORF is expressed together with nuclear-localized GFP (see recombination in Fig. 7d for the trunk and 7e for germ cells). We employed this system as being preferable to conventional overexpression because it allows us to bypass the deleterious effect of overexpressing the pleiotropic *sox5* gene during early development. Using this system, we show that in terms of germ-cell numbers, a partial rescue of the Sox5^-/-^ phenotype at stage 20 occurred in the *sox5*^-/-^ mutant genetic background (Fig. 7f). This provides evidence that Sox5 is indeed required for controlling PGC numbers during the formation of the early gonadal primordium as early as stage 20.

### Female-to-male sex reversal of Sox5 mutant fish

**Fig. 8:**
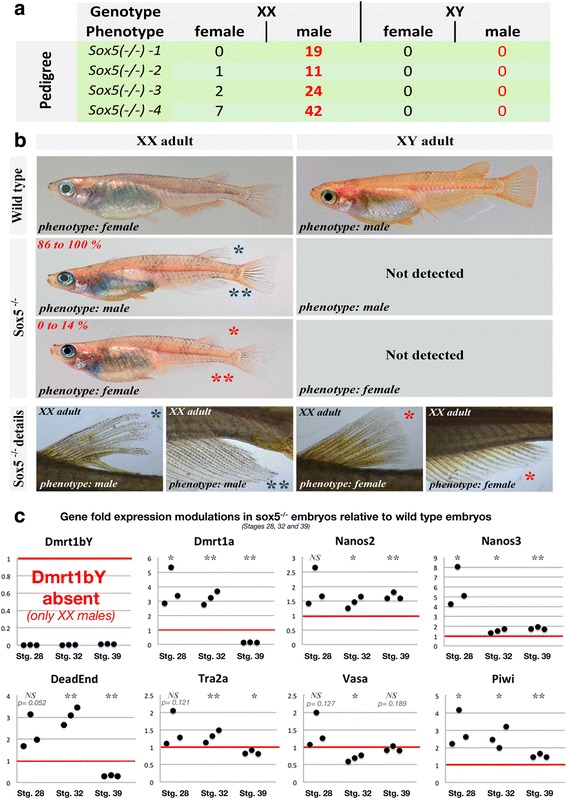
Phenotypic versus genotypic sex of *sox5-/-*mutants and regulation of the *dmrt1* co-orthologs and a set of germ-cell markers in *sox5-/-* mutant embryos. **a** Phenotypic versus genotypic sex of *sox5*^*-/-*^ mutant fish. Complete XX female-to-male sex reversion was obtained. **b** Sexual phenotype of the adult medaka. Wild-type XX females have a triangular-shaped anal fin as well as fused dorsal fin rays. Wild-type XY males have a parallelogram-shaped anal fin as well as split dorsal fin rays. **c** Regulation of the expression of *dmrt1bY* and other germ-cell markers (*nanos2*, *nanos3*, *dead-end*, *vasa*, *tra2a*, and *piwi*) in *sox5*^*-/-*^ mutants compared to wild-type embryos at different stages of development (stages 28, 32, and 39). Dataset results of three different batches of eggs obtained from different couples. Statistical significance was assessed with the *t*-test (*N* = 3 and each replicate is a pool of 25 eggs; * *p* value ≤ 0.05, ** *p* value ≤ 0.01). ns non-significant

Since germ-cell number was reduced in the *sox5*^*-/-*^ mutant fish, we further investigated whether sexual development was affected. Phenotypic and genotypic sex was determined in a *sox5*^*-/-*^ mutant line [31, 34]. A complete XX female-to-male sex reversal (up to 95%) was recorded (Fig. 8a). The female-to-male sex-reversed phenotypic males were fully fertile, so this *sox5*^*-/-*^ mutant line could be maintained exclusively on a XX genotypic background (see Fig. 8b for phenotypes). Genotypic XY *sox5*^*-/-*^ mutant fish were never detected in the progeny (Fig. 8b) nor in outcrosses. The absence of XY *sox5*^*-/-*^ male-to-female sex-reversed fish, predicted to overexpress *dmrt1bY* according to our results, is in line with the scarcity of surviving YY zygotes—carrying two copies of the *dmrt1bY* gene—reported in the literature [35].

Monitoring gene expression in the XX embryos of that line, we find the upregulation of PGC marker genes, including *nanos2*, *nanos3*, *dead-end*, and *piwi*, whereas *tra2a* and *vasa* did not show significant changes compared to wild-type embryos (Fig. 8c). Of note is the upregulation of the autosomal *dmrt1a* in the Sox5 mutant background. The precocious expression of this gene at early stages of development (stages 28 and 32 in Fig. 8c) is intriguing because *dmrt1a* expression is not expected before 10 days after hatching. This untimely expression may be related to the XX female-to-male sex reversal, because a similar untimely expression of dmrt1a has been seen in high-temperature-induced XX male-to-female sex reversals [36] (and our own unpublished data).

## Discussion

SD relies on the proper control of a hierarchically structured, multilayered network of genes. The genes at the top orchestrate complex transcriptional and post-transcriptional regulations (see [3, 4, 6, 37] for reviews). Despite such a critical function, they appear to be dispensable in evolutionary terms and can be quickly replaced with the emergence of new lineages. Our present analysis provides evidence that concomitantly to the acquisition of a dominant position within the SD network, the medaka master male determiner, *dmrt1bY*, was subjected to a profound rearrangement of its regulatory landscape. We found that sequential insertions of both *Izanagi* and the *Rex1* transposon were instrumental for rewiring the *dmrt1bY* promoter in the process of diversification from its autosomal progenitor *dmrt1a*.

First, the integration site itself appears to be highly relevant. We have previously reported that a *P*-element-like DNA transposon, *Izanagi*, brought in a regulatory sequence that mediates specific transcriptional regulation of *dmrt1bY*, which was important for the Y-chromosomal duplicate to evolve its new function [17]. A common feature of class II transposons is that they can excise. The insertion of the *Rex1* element in the *dmrt1bY* promoter occurred in the DNA-binding domain of the transposase and thereby fixed the *Izanagi* element and the contained Dmrt1 transcription factor binding motif to the promoter of the new SD gene.

Second, the *Rex1* transposon contributed a functional high-affinity binding site for the transcription factor Sox5 as a novel regulatory element for *dmrt1bY* expression. Thus far, neither Sox5 in vertebrates nor its *Drosophila* homolog Sox102F [38] have been shown to be implicated in SD. In medaka, the expression pattern of *sox5* already indicated a function in gonad formation. Indeed, independently of *dmrt1bY*, *sox5* is expressed in the lateral plate mesoderm that later gives rise to the gonad. Thereafter, during gonadogenesis stages, *sox5* expression switches toward the germ-cell lineage. There the *sox5* expression pattern in PGCs is mutually exclusive with the expression of the master SD gene *dmrt1bY*, which, during the formation of the gonad primordium, concurrently changes from germ cell to somatic cell expression during this period. This is consistent with the in vitro findings of a suppressive action of Sox5 on the *dmrt1bY* promoter, although the early expression of *sox5* in the lateral plate mesoderm as well as in the adult gonads suggests other additional gonadal functions de-correlated from *dmrt1bY* activity.

It has been shown that Dmrt1bY has a suppressive effect on cell proliferation by mediating a G2 arrest [39]. Thus, continued expression of *dmrt1bY* in PGCs during early embryonic stages, which precede the actual SD stage at hatching, could have a negative effect on the number of PGCs that is presumed to be decisive at the SD stage [40, 41]. Clearly the observed suppressive action of sox5 toward *dmrt1bY* expression in vitro and in vivo at stages 33/34, down-modulates this negative effect.

Later, the persistence of *sox5* expression—independently of *dmrt1bY* expression—in germ cells within the gonadal primordium and in the early-differentiated germ cells of mature gonads indicates another independent major role in germ-cell physiology from gonad induction to adult development and maintenance. Hence, it is likely that following the transcriptional rewiring of *dmrt1bY* first by *Izanagi* and then by *Rex1* TEs, Sox5 has been hijacked in the primary SD cascade for controlling and fine-tuning *dmrt1bY* expression during the male-determining period. Independent of the regulatory function of *dmrt1bY*, Sox5 appears to have a more general involvement during gonadal formation (visible by expression in the lateral plate mesoderm) and germ-cell physiology (apparent from persistence of *sox5* expression in germ cells).

Although not directly related to *dmrt1bY* regulation during the formation of the male gonadal primordium, the most convincing evidence for a sexual development function of Sox5 comes from medaka strains that carry knockout alleles for this gene. We find that lack of Sox5 leads to a decrease in PGC numbers, which is rescued by re-introducing the wild-type version of the gene in mutant embryos. Strikingly, at the molecular level, we found an upregulation of several germ-cell markers in the mutants, even though the germ-cell number is sensibly reduced. It can be assumed that such overexpression in mutants is an indication of an insufficient compensatory mechanism needed to rescue germ-cell numbers properly. These findings demonstrate that germ-cell marker expression levels upon Sox5 modulation are primarily the result of gene expression regulation and are not due to the number of cells that express these genes.

The XX female-to-male sex reversal in the Sox5 mutant is in line with an inferred important function of maintaining the appropriate number of PGCs. The number of PGCs at the SD stage is critical for determining male or female sex in medaka [39, 42]. It is higher in females at the SD stage. Lowering the number of germ cells in medaka or zebrafish results in female-to-male sex reversal [43, 44]. Hence, when the number of PGCs falls below the threshold in sox5^-/-^ mutant XX fish, it will permit male development. Interestingly, we find an ectopic and earlier than normal expression of *dmrt1a* in the primordial gonad of the mutants. This has also been observed in environmentally induced XX female-to-male sex reversal in medaka and has been interpreted as a compensatory mechanism to supply the necessary trigger for testis development in the absence of *dmrt1bY* [36, 45].

Sox5 regulation of *dmrt1bY* and the importance of this gene for sexual development in medaka raise the question of whether this co-option of Sox5 regulation through *Rex1* insertion brought a novel member into the SD regulatory network as a medaka-specific evolutionary innovation or whether this event provided a necessary connection to an indispensable gonad-development downstream pathway. This is difficult to answer at present but should motivate further studies on the role of Sox5 in the formation of ovaries and testes in other species.

The in vitro data of *sox5* effects on *dmrt1* transcriptional regulation in zebrafish and wrasse [18, 19] point to an evolutionarily conserved function of Sox5 that have been unnoticed so far. Members of the SOX family of transcription factors play essential roles during SD in mammals. Both the founding member of the SRY family and the closely related factor SOX9 have been shown to be necessary and sufficient for mammalian male SD [46, 47]. In addition, other SOX family members, such as SOX3 and SOX10, can take over this role if they are expressed ectopically in the developing testis at the time of SD, as demonstrated in transgenic mice and in human patients with duplications in these genes [48–53]. In contrast, *Sox5* has been implicated in spermatogenesis in the adult [54, 55] but not in embryonic gonad development and/or SD in mouse. With all necessary notes of caution, our preliminary data detecting SOX5 expression in the fetal gonad of mice may indicate an additional role for SOX5 during embryonic gonad development in mice after SD.

The finding that a preformed transcription factor binding site contributed by the *Rex1* transposon modulates the regulation of *dmrt1bY* promoter highlights the important role that mobile elements play in the genome for shaping the evolution of new functions. Intriguingly, although bona fide examples of this process are still rare [16], *Rex1* is the second such event found in the same promoter. It will be interesting to analyze whether the other repeats present in the *dmrt1bY*, but not in the promoter region of *dmrt1a*, provide further instances of TE exaptation. Genes that arose by gene duplication such as *dmrt1bY* are primarily dispensable and can only escape degeneration through sub- or neo-functionalization. As *dmrt1bY* and *dmrt1a* both have exclusive functions in male sexual development in line with the highly conserved role of *dmrt1* in invertebrates and vertebrates [56], a change in transcriptional control via the insertion of two different TEs might initially have led to sub-functionalization; *dmrt1bY* acquired its transient early expression, whereas the transcription of *dmrt1a* was pushed back to the later testis differentiation phase. In other fish species, and in mouse and chicken, *dmrt1*, which represents the evolutionary precursor of the two genes in medaka, is expressed starting in very early male SD stages and continues to be expressed during testis differentiation and specialization and in the post-pubertal reproductively active organ [6, 37, 57–59].

## Conclusion

In summary, the evolutionary history of the promoter of a newly arising SD gene in medaka not only provides a new example for TE-mediated rewiring that created evolutionary novelty but also shows the unexpected complexity and richness of such elements. It will be interesting to have a closer look at the SD genes of other fish that have been subject to fast evolutionary change and thus, might also be targets for TE exaptation.

In addition to showing that *sox5* was recruited—or more exactly promoted thanks to neo-functionalization—to the very top of the primary SD cascade after insertion of *Rex1* and that it controls the fine-tuning of *dmrt1bY* expression, our results provide evidence for a more general and ancestral SD function of Sox5 in regulating germ-cell number and, in consequence, gonadal identity.

## Methods

### Immunohistochemistry

Testes from adult fish were fixed with 4% paraformaldehyde in balanced salt solution (111 mM NaCl, 5.37 mM KCl, 1 mM CaCl_2_^.^H_2_O, 0.6 mM MgSO_4_^.^7H_2_O, and 5 mM Hepes, pH 7.3) for 30 minutes on ice. After fixation, samples were washed three times for 10 minutes with MABT buffer (100 mM maleic acid, 150 mM NaCl, pH 7.5, and 0.1% Triton X-100) and subsequently twice for 30 minutes with MABDT buffer (MABT buffer complemented with 1% bovine serum albumin and 1% dimethyl sulfoxide). After blocking in MABDT-blocking buffer (MABDT buffer supplemented with 2% lamb or sheep serum), the tissues were incubated in MABDT-blocking buffer together with anti-11-β-hydroxylase primary antibody (1:150 dilution) overnight at 4 °C. Samples were then washed three times for 5 minutes in MABDT buffer and washed again four times for 30 minutes in MABDT-blocking buffer on ice. Thereafter, samples were incubated overnight at 4 °C with the secondary antibody diluted at 1:600 in MABDT-blocking buffer. Finally, the tissues were washed in phosphate-buffered saline, stained with Hoechst solution for 3 hours at 4 °C, mounted and imaged with a confocal microscope (Nikon C1 confocal microscope). For Additional file 4: Figure S3, immunohistochemistry was performed according to [60] using the mouse anti-SOX5 antibody from Abcam (ab26041).

### Chromatin immunoprecipitation

For ChIP, the EpiQuik ChIP kit (Epigentek) was used according to the manufacturer’s instructions, using 1 million cells (Sg3 spermatogonial or OLF fibroblast cell lines) of transiently transfected cells with FLAG-tagged Sox5 and 3XFLAG antibodies for immunoprecipitation. After fixation and cell re-suspension, DNA was sheared by sonication (nine pulses of 10 seconds with an amplitude of 10%). After immunoprecipitation, specific primer sets were used for enrichment quantification by real-time PCR. For controls, primer sets encompassing regions without any sox binding sites were used. The results are presented as enrichment compared to input. All primer sets were checked for the specificity of the amplifications.

### Bioinformatic analyses

Binding sites for Dmrt1bY were identified using the matrix provided by [61] together with the Regulatory Sequence Analysis Tools portal (RSat) [62]. Sox5 transcription factor binding sites were determined using MatInspector from the Genomatix portal [63] using the following positional weight matrix: [A, C, G, T: (4, 6, 3, 9), (7, 4, 3, 8), (21, 0, 1, 1), (22, 0, 1, 0), (0, 22, 0, 1), (23, 0, 0, 0), (22, 1, 0, 0), (0, 0, 0, 23), (10, 3, 6, 4), (5, 7, 6, 5)].

### In vitro expression regulation analyses and real-time PCR

Medaka spermatogonial (Sg3) and fibroblast-like (OLF) cell lines were cultured as previously described [64–66]. For transfection, cells were grown to 80% confluency in six-well plates and transfected with 5 μg of expression vector using FuGene (Roche) reagent as described by the manufacturer.

Total RNA was extracted from fish tissues or transfected cells using the TRIZOL reagent (Invitrogen) according to the supplier’s recommendation. After DNase treatment, reverse transcription was performed with 2 μg of total RNA using a RevertAid First Strand Synthesis kit (Fermentas) and random primers. Real-time quantitative PCR was carried out with SYBR Green reagents and amplifications were detected with an i-Cycler (Biorad). All results are averages of at least two independent real-time reactions. Error bars represent the standard deviation of the mean. Relative expression levels were calculated (according to 2 – ^ΔCT^ where CT is the number of cycles) after correction of the expression of elongation factor 1 alpha (ef1alpha).

### Luciferase assay

For promoter analysis, a 9107-bp fragment upstream of the Dmrt1bY ORF was isolated by restriction enzyme digestion (*XhoI/EcoRI*) from BAC clone Mn0113N21 [17] and cloned into pBSII-ISceI plasmid (pBSII-ISceI::[0/-8927] Kb Dmrt1bY. Subsequently, the Gaussia luciferase gene from the pGLuc-basic plasmid (New England Biolabs) was inserted between *EcoRI* and *NotI* sites of pBSII-ISceI:: [0/-8927] Kb Dmrt1bY prom (pBSIIISceI:: [0/-8927] Kb Dmrt1bY prom::GLuc plasmid, Fig. 2). pBSII-ISceI:: [0/-1593] Kb Dmrt1bY prom::GLuc, pBSII-ISceI:: [0/-2963] Kb Dmrt1bY prom::GLuc and pBSII-ISceI:: [0/-6162] Kb Dmrt1bY prom::-GLuc plasmids were constructed in the same way, removing 5′ fragments of the 9107-bp Dmrt1bY promoter region using *Kpn1*, *Eco47III*, and *HindIII* restriction enzyme digestion, respectively, and re-ligation.

Gaussia luciferase activity was quantified using the Luciferase Reporter Assay System from Promega and normalized against co-transfected firefly luciferase expressing plasmid (ptkLUC+). For Fig. 2c, [*α*], [*α*]-MUT, and [*β*] fragments (same as used for the ChIP assay shown in Fig. 2a) were PCR amplified and cloned into ptkLUC+ plasmid (accession number AF027128) between *HindIII* and *BamHI* restriction sites.

### Establishment of transgenic reporter lines and in vivo recombination and imaging

A transgenic line was created for the in vivo visualization of endogenous sox5 expression as well as in vivo functional knock-in of sox5. The Sox5 upstream promoter region of the Sox5 gene was cloned in front of an [mCherry-stop] cassette flanked with LoxP sites (Fig. 7c). In detail, in a first line, the sox5 promoter region drives the expression of an mCherry-Stop cassette. This cassette is followed by a Sox5 ORF fused (2A self-cleaving system [33]) with a nuclear-addressed GFP (H2B-GFP). For recombination, direct microinjection of one-cell-stage embryos with mRNA encoding for the *Cre-NLS* protein was performed (Fig. 7d–f). Sox5 ^-/-^ homozygosity of the fish was determined according to the pigmentation pattern of the embryos (see [21]).

To generate stable transgenic lines, the meganuclease protocol was used [67]. Briefly, approximately 10 to 15 pg of total vector DNA in a volume of 500 pl injection solution containing *I-SceI* meganuclease was injected into the cytoplasm of one-cell-stage medaka embryos (Carbio strain). Adult F0 fish were mated to each other and the offspring were tested for the presence of the transgene by checking for fluorescence. Siblings from positive F1 fish were raised to adulthood and tested again for fluorescence.

For PGC visualization and counting, the GFP-nos1 3′ UTR construct that includes the mmGFP5 ORF cloned upstream of the 3′ UTR of the zebrafish nanos1 gene [68, 69] was injected at the one-cell stage (Fig. 7f).

For imaging, embryos, hatchlings, or tissues were mounted with 1.2% low melting temperature agarose. Confocal pictures and image stacks were acquired using Nikon C1 (eclipse Ti) confocal laser scanning and the NIS element AR software.

### Whole-mount in situ hybridization

RNA whole-mount in situ hybridizations were performed as previously described [70]. Hybridization signals were detected using alkaline phosphatase conjugated anti-DIG antibody (Roche) and BM-purple (Roche) as chromogen.

### Bioresources and animals

The Sox5 medaka mutant strain (N541S) has been deposited with the National Bioresource Center [71].

## Additional files

Additional file 1: Figure S1. Annotation of the *dmrt1bY* promoter. Bold underlined: *Rex1* element. Bold red: location of sox5 binding sites. *Dmrt1bY* exon 0 is in blue letters. Primer sets used for ChIP are provided. (DOCX 169 kb)

Additional file 2: Figure S2. Comparison of medaka Rex1 reverse-transcriptase (RT) sequence with other non-LTR retrotransposons. RT conserved domains are given according to Malik, Burke, and Eickbush [72]. RT sequences are CR1 from *Gallus gallus* (U88211); Maui from *Fugu rubripes* (AF086712); Jockey, R1, and I from *Drosophila melanogaster* (P21328, X51968, and M14954, respectively); and Tad1 from *Neurospora crassa* (L25662). The degree of amino acid conservation between sequences is shown at the foot of the alignment. (JPG 811 kb)

Additional file 3: Table S1. Location and adjacent genes of *Rex1* elements containing Sox5 binding sites in the medaka genome. (PDF 70 kb)

Additional file 4: Figure S3. SOX5 protein expression in fetal mouse gonads. Double immunofluorescence of SOX5 (green) and MVH (red) on sagittal sections of 13.5 dpc (left panel) and 14.5 dpc (middle panel) mouse testes, as well as 14.5 dpc mouse ovaries (right panel). Gonads are demarcated with dotted lines. The lower panels are a higher magnification image of the area marked by a square in the upper panels. Scale bars 100 μm (upper panels) and 30 μm (lower panels). (JPG 1710 kb)

Additional file 5: Supporting data. Raw supporting data for Figs. 2A,B1–B4,C, 3A,B, 7 F, and 8C. (XLS 215 kb)
