# Supplementary material for: Sox5 is involved in germ-cell regulation and sex determination in medaka following co-option of nested transposable elements
Source: BMC Biol. 2018 Jan 29;16:16. doi: 10.1186/s12915-018-0485-8 (PMC5789577; doi:10.1186/s12915-018-0485-8)
Supplement: Supplementary file 1 — Annotation of the dmrt1bY promoter. Bold underlined: Rex1 element. Bold red: location of sox5 binding sites. Dmrt1bY exon 0 is in blue letters. Primer sets used for ChIP are provided. (DOCX 169 kb) [file 12915_2018_485_MOESM1_ESM.docx]

>dmrt1bY promoter

CTCGAGCCAGCCAGGTGAGACGTTGAAGCTCTCCACCAATCAGGTAGCAGCTTAACCACGTTTCACAGCAGAACACAAAGCACGTTGACATTTAAGCAACACCTGGTGGTCAGGCAATCCTTGTGAATGCTGTGGTTGAGGTGTGGGACGGGTACCGTTTGAATTTCAGCCGGTGCTAAACCGGTCCTTTGGAAACGGTTTTGGCAGCTGAATGCGCCCGCATCGGTGAAAACGCTTGACTTTTTCCTGTAAACTGAAAAGTGCACCAAAGCAGCATGAGGGACAGCCTTTTTTCATCTATGTGGTGCTCGGATCCATCAGAACCGTCTGATGTGAAGTTCGGCAGAACCTTTCATTCAGTGTTCTCACTGAAACTCCGCCATCAATAAGGCCAACCCATTCCTATATGAGCCCGCCTACAATCTCCAGGAATTAAAAATGACTGAAGCAAAACTGCACAGGAAACAAACAATGGACGCGTCGGGAATGAAGCACGATCGTTGTTTTTTGTGGTCTGGTGGTACTGAATGCATCTGTAATGGTACCACTGCTGGGTTCTGGACACTGAGATGATGAAGATGCTGAAGGTGATGAAGATGCTGAGGTTCTGGATGTGATCAGGAAGAGTCCATCAGTGATCAGATCATGGTGAATGTGCTGCAGATCAGAGCAGGGAAGCAGATGGAGGAACAGGGATGAGTATGCTGACAGGATTCTGAGGAGAGACCACAGAGGAGGTTCATGGATGAGGGAGGAGCAGGAGACAGAATAGGAAAATTATTCTAAATAACCAGATCTTCATCCTCATGTGTCTGTTTTGCAGGGAATGCCTCGGCTCAGCAGGAAGGCTTCGCCGAGCCCCACCCGCAGAAGTGTCTTTGATTAGCCCCCCCATTCCACCTTGAGGTGCTGCTTTGGCCTCAAACCACCAATGAGATTTCTACAAATGTTTGCCTCCATGAGGAGCCCAAAATGCCCTTCAGATCCTCCTTTTGTACTGTTTTCTGCTGTTCTGTGACCAATCCGGAGTTTTCTGATCCCCCCCCCCCCAGTGTTGAATGGGCCATCAGACCGGCTCCACAGAAGTCCTCCGTTTGTATTTACAGAGAATCTCAGTATTTTGAATATTTATCAGAGTCTAGTTTAATGCAGAGATCCTTTCTCTGTACAGATGCTGCTCTCAGTCTGCTGCCCTGTAATACACGGCGTCTGTCCTCTGAGGTTCTAGAATGTTCTGGAAGGTTCTGCTGTTCGTCTGAAGTGCCTTCACTTGTTTTGTGACAATAAACTTTTAATAAAGTGTAAACCCATGTGCTGGTGGTGGTTCTGATCCGTCTGTAAGGAGGAAAGGAAAACCTTAGAGCAGCATAGGATCTTTGAAGGTCAGCATTTAGAACGTTCTGCACTTCTTCCAGGTGCAAACCCGTCTTTGGTCAGCTTCGTTTAGAAAAACTAAAAATCAGGTCTCTCACCTCAAAATATCATCTGAAGTCAGCTTCAAGCTGATATTCTGAGCAGAAAAAAAGATTTTCAGTGAACACAGAAGCTCTGAACTGATGACAGGTACAGGTCTGATTAGAACCTGGACCCTGCTGAGCCTCATGGTGCCGTGAGTCCAAATCCTGTCTGAATGTCATCAGAACGCCCTGCAGTGTACGCGAACAGGCAAACTTCCACTGGAGTCCCTCCTAAAGTCTGTTGGAGGTTGCCATGGAAACATCATTCACTTTAATGTGAGTCGCTCGGACTCCTCTTCAAACCTTTCTCTGACGGTTCTGATACGTTCTCCTCAGGAGTTCACCTCCTCTGAACCTTCGCCGCTTCTCCGTTTGAGGCTGATCACACTTGGAGAAGAAAACAGTCTTTTCTTGCGATGGAATGAAGACGAAAGCTCAGCTCAGACGTAGCTCAGACCTCTGTCACTTCTGATGATCAAAGTTTCTGAGAAACCTTTTTCCCCCAGAATGCATTACTGTGTTTGCTAAATTTGGAGGTTATTTCTGCACAAAACTTTAGATTTCAGACCATTTAAGTTTTTCCAGCTGGAGCTGCATGACGGAGCGAGAGCTTCACGCCTGCTGCATGACTGCTCTCCTGCAGGAACATGGTGAGAAGAAACTGGGTCAGAACCTGCAGTTAAGTCTGGTTCTTCACCCTGAACCAGCTCTTAAAACCTCCAAACTGACCTGTGGTGGAGTGGCGAGGTGCTGGTTCCATCTCCCAATCGGGACGTTGGGGTTTTGTTTGATTGGGGGGTGGGGGGTGGGGTGTGTGTCTGGAATCTGGGATTTGGTTCCCATCCTGTAAAACGGGACGTCGGTCTGCGAGAGCAGCTTCCGTTGGACATCCGAGTCGCGTTCAGAGTCCAGATCCTGCAGGGACAGAACCAGCAGCGGTTTTTGAGCTGACTCAGAGAATGCAGTTTTCTGCTGAGTCGGGTTGGAGACCGGTGGCTGCCTCCTGCACGGCGCTCCTCATCTGCCGCCGCCTCCTTTCTTCCCTCAGCTCTGCCTCCATGTGGCCCATCTGGGCTCGGAGTTGACCACTCTGCGGCAGCCGTTCCGGCGCTGCCTCCTGGATCTACCTGGGATGGATCTACCTGTTCATGACGCTTTGTATGTTTTCTCTCTATACCAACAGCCATTGGGATAGGCTCCAGCAACCCTGCAGGGACATGGCGGGTTTGGAAAATAGACGGTTGTTCCAACAGTAACATGGAGGGGGGTGTAAACACTTTTTGGTACCAGTCCAGTTGTTTTGGCAGAAGCGCTGGGCTCTGACCTCTGAACCTGCAGTCCGTCAGTCCTTGCTGCAGGGTGGGGACGGTTCTTCCCCTTTATATCAAACAACACAAATGTTAGAAAAAAGGTCCAACAGGAGTTTAGACTCATTAAAGGCAGGAGTGTTTTCTCTGATCGCAATGTTGTTCTTCGTCTTGATAAGACGATAAAATAATTGTAACACTTTCGTGGATATTTCTCACACGAATTATCAGAGAAAATCTGAGATTTTCCCAGAAGAGGATGTACCAGAAATGTTTCCTCAAGGCAGGCAGAAAAAAACCAGAGCCTTTCTGCTTCACTTGACATAGAGGTCAAACTGCACCTGTTAAAGATAGCGGTTCAATAAACACTGCCAGGAGCACGCCAGCATGGCGGAGGGGCCATGATGGGGGTCGTTTTTTTGGGGGCTCGGGGGCCGTCAACTTGAACATCTCAAAGCAAGTGATGCCTTAAAACCTAAAGAGGTTTGAAACCCAGAAAAGGAATTGTTGGCCTCCCATCCGGCCTTCACAGATTCAGGTGGTTCAGGACGTCCAAAAATGAAATGTGCAGTTGGGAAATGAAAAGACACACAAGAGTCTGCATGAATTTTAAAACCTTTGCAAAAGGGGAGAGGTGGAAAAACACGACCTGTTAAGCTCTGTGGCTGTCCGGTCTCTCTGTCCTCAAGGCAAAAAGGTTTTTTTTATTATCCAGTAAGGGAAGTTGAAAACATACAAAATACGTATTAGAATGGTGTGTTTTTGTCTAGTCTGCATTGCAGGTGTTGTGGCTCCATAAACACTCCAGTACAGAAAGAAAACAGAGACCACAAGTCAAAAACAGCCTTACTGGGGACCTCGTCAGATATTATTGCTACTGAGAGCAGGAATCAAGCATATTTCTGCAGTACAATATACTCGCATATGTAGAATCCATTAAATGCATAATAACAAGAACATAGAAAATATAATAGAACCCCTGTTCACCTTAACAGAAGCAATCTGCAAACTGGGTTCTGACTTTCAGCGTCTCACCTGGACTCACCCTGGGTTCTGGACTGACTGATCAGGACCGCCGTTTAAGTCCAATCGCTGGATCAAACCAAGAGTGAACAAATTTGTTAGTATTTTATTGTGAAGCAGTAAAACATCAAAATAGACTTTTGGTTGTGCATGGTGGTACAGTGGTTGATGCCCCAGCCTCACAGCTGGAAGGCCCTGATTTGAATCCCAGCTGGGTATGTGCATGCATGGTGTCCCTGCAACAGTCTGACTATCTGCTCGCCTCCACCCAACAGAGTAGCCGGGATAAGCTCCAGCAACCCTGTGACCCAGACGGGTCTGTGGGGTCTGGGGATGAATGGATGGAGTTGATTTTTATAGTAGATGGTTCAAAGATCCCACCTGATCTCTTCTGTGGTACTTTAGTCTGGATCCTCTGCCATGCCTCTTCTGACAGATTTGGACCAGAACCTCATCTCACAGGAGACTGTGTTGTGAGGCCCTCGGATCCTCACAGGAGGTGATGGCTCCTAACAGAAGCCCAGCTGTTCTCATGGCAGATTCAGCAGACAATTTGCTTCTCCTTTCAGTCCCATCAGCTTCCTCTGATGCAGAGGAGGAGTTTCTCCTCTGAGCTGTGGGTGGGAGACTGAGGTCAGATTTCACTGGAGCAAACTGCTTTCTGGTGCTTCAAACTATTTTTATTCAATGAAATGTATATATATATATTTCTCTGCTGCAGATGATGAGCTGACCAGCGATTAGCCGACTGTAAAGCCCTGTGAAAGTTGTACCTCCTCAGAAACTCCAAAACTGTTGTTCCCGTCTCCAAACCTCTCGTGTTCCAGCAGGTTCTTCATCTCCAGGCCGTGAAGCTCATCTCTGTTTTCTTCTCCTGTGCTTCAGCTGCTTCTGCAGGGAGGCAGTGGTGGAGGTCAGCTGGTCTTTGTGCTCCAGCTGCTCCTCTGTGAGGACAGGAGTCTCTTTCAGCCTGTTGTGTCACACCGTCTTCTTGGAGAAACGACAGCCAGGTCAGCGACGTTCAATCTGATGTGTCAAAAGATCTTAAAATGCTCAGGATTTTCAGAAGTAGCAGATGCAGAAGATCTTACTTTTTTTTTTTTTTTTTACCAAACTTTCTCCGCCTACAGGGAGAAGGTCTCACCAAGCTCCCTGTTTCTCTGCGTGAGTTCCTGGATTTGCTCTTGGAGCTCCAGACTGAGCTGCAACTGCTGCAGAGCGTCTCTGTCGTTACCTGCAGCCTCCTCTGAAGCTCCAGCTGCTCCGACAGCGCCTCCACCCGCTCCTTCTCCTCCAGCAGGGAGCGATTCAGATGCTCGATGATGTCGTTCTTGTCCTTCTCCTCCTCCTCCTTCTCCTCCTTCAGCTGAAATTGTAGGAGGATGTTAAAAAAACACGTCTGAATATGTACGTAACATTTAGGTTTATTTACATGATGCTATGTTAAACTAACAAAAAGAGAAAATAATAACTCATTCAAAGTCACAGTCTGTTCCATTGCTGTATCTAAGCACTGGACTGAGTGACGTCCTCAAATGGTTTCTTCTGCCTTCAACCAAATGAAGTCAATTCTGTTGAAATTGCTTTCAGATACAGACGCTGGGGGCTAGACGTCGTGAACATGCTGGAATTGGTTGGGCTTAACGTTTCTAAGGCAACCACTCTGGGCAATCAGGGGTGAGCTTATTAGAAGCCCACACCCCTACCTCTTGAAAATAGGCTACATCAAATCTATCAAACACTTTGAACGTTGGAGTGGGGGCCAACCATTGACTGTATGTGAGAACTGGACTGAGTGACTCCTCCCCCCTGGGGCTCCAAACAGGAAGTGGTTCCAAAATGCCCAAATCCCACAGACTTCTATAGAGAAACTACAGCTGTCCTCAGTCATTCAGAAGAATCGTTCTGGATCTGCAACCATCATCCTGATAATTCTCTGTTTTTTCAGGATATTTTCTCTGTAGTTCAAGTTGTTTATGTTAGAAACTGACCAATTAGATGCCTCAATAAAGTAGGTGGAGCCTGCTGCCCCCACATCCAACGTTCAAAACAATTTATAGAGTTGGTGTAGCCTGCTTTGAAGTGGTAGGGGTGTGGCTTTCCAACAAGCTCACTCCTAATAGGTGAGAATTGTTCTATAGAAACATGAACTCAGACAGACTCGGACTGTTTAGTTAAAGCTTTTACCAATCATACCTCTCATGCCATGTTATCATCGTCACATAAACAGTAGGATGCGGTTGGGATAATAGACAGTAATGGCCGGCATCTCATTTCTGACACCATATGAGCACATTATAAAACTGTAGCGACCTGGGGGTTAGCCCCGCCTTCAGCCCCTAAGATTCGTGGGAAGTGGGGAGCTTAGGGTGAAATCTTGAGTCAGAGGCTGGAAGCTGAAGTGACCCTCAATGCTGTTTGGGCTGTATGTGTGCTTGAATAAACGGGCTTGATGCTCAAAAGGAGACATTGCCTTTCTTCCTGTTTATTTCTGCCTGAGACTGTCCGGGGTCGTGGGTGTATGGGGCACGGACAGCTCTCCAATGAAGCCATCTAAATAGCAGTGTGGCTTTTGCTTCCCCACTAAACTGACGGTCCGGCTGGATCGTTACATTACTTAGATTTTTACTTATTCGCGTTTAGCAACGTTTATGTTGTAATCAATTGTGTGTTTTGTGAACTAAGAATATGGCAGCCAAGAGACTTCCTGCCAGTGACTTCCGGTCTCTCCGGCGTGTGGCTCAATGAGTTATATCACTAGAGGAGACACCCCGTTTTAGAAGCCGGGCCAACGGTGGCGGAGCGCGACACTATCTTGGTGAGGTCGACGATGCCCACATGACAATGATTTCTATTGCTTTTAGAGGTGTCTAAGTAGCTTTTATATACTATATATATTTTTAAATTTATATATATTTACACATATATGAAATATATTTATATATATAATATATATATAGATACATCATTTGTTGTTGTTAAAGAAGAGAAACAGTCAGCCTTAAAAGCTCAAACTTTAATGAAAATGCATTGTTGCAGGTCCTCTTACAAATAAAAAATTACTACAAAGAATTTTGAAAAAAATAATTTGTAAACATTTAAGAGTTTTAGACCTCTTTGACATGTAATTTAAATTATTTAGAAAAACCCAACAATAAGTCAATAATAATAATAATAATAATATAGTAAATCAATAGTAGGTCAATACAAACAATAAATAATAACAATACATAAATAATAAGTCAATAAATAATAATAAAAATAAGTCTATTAATAATAATAATAATAATAATAATAATAATAATAATAAAATAATTTTAATAAAAATAAAATAAATATTCAAAAGAAGTCCAAAAGTGAACAATAGATTTTCCCTTAAAAAAACAACAAAAAAGAAAAACAATAGATTTATACTGATTCATGTGAGTGATCAATTATGTAACACAGATGTTATGGTGTCGGGTTTCAAGCAAACAGTCTGGCCTCTTATCTAAATGTGTTTCCACAAATGTTCTGAACATAGCCAGACTGTATTACTAGTT***GGTACC****ACAGTGATCCATCTGG*TTAGGTCTTTTTATTGCTAAGATCCACTTTTTCCTTAAGTCTGAATC**CAAATTCAAATTCAAATTCAAATTTTATTTGTCACACACACAATCATACAGCGTACAATGTGCAGTGAAATGCTTAGGCGCCGCCCGCGACCTCAAACAAGAAAGAATATCAATAGGAATAGGAAGATAAATATAAAATATAGGAATATACAAATATAAAAACATAAAAACAGAATGTCCGAATGTGTGCAAATATGCTCTAAAAGTACATGTGAAAGTGGCTTGTGTGTATAATGTCCATGATTACTGGTTAAGAGTCCGTATCGCCTGCGGGAAGAAGCTCCTCCTCAGTCTCTCTGTGTTAGCTTTCAGAGAGCGGAATCGCTTTCCTGACGCAACAGCGAGAACAGTCCGTTGTCAGGGTGGCTGAGGTCCTTTGTTATCTTCCTGGCCCTGGTCCAGCACCGCCTGCCGTAGATTGAGTGCAGGTCAGGGAGCTCGGTGTGGATGATGCGCTCAGCGAACGCACCACCCTTTGGAGAGCTCGTCTGTCCTGCATGGTGCTGTTCCCGAACCAGGTTGTGATGTTTCCCGTCAGGATGCTCTCTATGGTGCAAGAGTAAAAGTTGCTAAGCACCACAGAGGGCATCTAAAGTCTCTCAGGCGTCTAAGATGGTAAAGACGCTGCCGGGCCTTTTTCACCACGGTGTTGATGTGACAGGACCATGACAGGTCCTGTGTGATGTGGACACCGAGGTATCGGAAGCTGTTCACTTCTCCACTGGGCTCTTGTCGATGACGGGGGTCTGGTAGTTCCTCTTCTGCTTTGTGGCGAAGTCCACTATCAACTCCTTTGTCTTGCTGACGTTTAGGAGGAGATTGTTCCTCTCACACCAGTTTTCA GATGTCCAATCTCCTTCAGGTAGGCCGTCTCATCATTATCAGTGATCAGGCCCACCACAACGGTGTCGTCAGCGAATTTGATGATGGTGGTGGAGTTGGTGGACGCCACGCAGTCGTACGTGTACATGAGTACAGTAGAGGGCTCAGAACACAACCCTGAGGATCCCAGAATC**CATTGGAAATCTGGAAGAAAAGTTCAGTCTCTGAGTCAGAAATGTATAAATCTATATTACTATAATAGCCTAGTATGCTGATACAGGTTAAGCAAAATAAAATGTACATTTATGTCGGCAGCAAATTATACGTACATCATGAAGCATGTTGATAACAGCCCTGCAGCATCATGCATGTCAACATGTGTATGATTAATGAAGCCTCTGAGCATTAAAAGAAAAAAAATTATAGTACTTACCTGTGAAATGATATACCTTCCTCCTTCATGGACTCAGTTCTCTGATTTTTGGAAGTTGAAACCCGCACAATGAACTGGTATTCTGCAAAAAATGTTTCACATGCATACACTGCAGCAGTAACCTGACCTCACCAAGATGGCGGTGATCTCCTCCCATGAGGAACCACGTGATGTCTCCTCTAGCGATATAACTCATTGGTGTGGCTGATTCCAGCTAATGACAAACAAAAAGGGGGGGGGGGGGGGAGTCGGAGCCAAGCGGGTACAACATTCT(“0”)GAAAGACTGTTCTCCGGTAAATTGACGCACAGCATCTGGCTTCACCGTTGGAGGATTTAACGAAACTCTGCAACTACTGGAAGGTATTCCCTTTGACCGGAAAGTCAGACGAATTC (“+117”)

Primers for cloning regions into ptkLuc+

Beta_Hind_Fw: AATAAGCTTGAGCGCGACACTATCTTGGTGAG

Beta_Bam_Rv: AATGGATCCAAGAGGACCTGCAACAATGCATT

Alpha_Hind_Fw: AATAAGCTTCGGAAGCTGTTCACTTCTCCAC

Alpha_Bam_Rv: AATGGATCCCCTGAAGGAGATTGGACATCTGA

Alpha_Mut_Bam_Rv: AATGGATCCGAGATTGGACATCTGAAAACTGGTGTGAGCCTCCTAAACGTCAGC

CtrPtkLuc_Fw: TGAACCTGAAACATAAAATGAATG

CtrPtkLuc_Rv: GCTCGGTGTTCGAGGCCACACG

Kpn1Mut5’Fw: GTT**GGTACC**ACAGTGATCCATCTGG

Not1Mut5’Rv: AATGCGGCCGCCTCCTAAACGTCAGCAAGAC

Not1Mut3’Fw: AATGCGGCCGCTCACACCAGTTTTCAGATGTCC

BamH1Mut3’Rv: AATGGATCCGAATTCGTCTGACTTTCCGG

CtrGLUC_Fw: CTGACGTTTAGGAGGCGGCCGC

CtrGLUC_Rv: GTTGAAGTCTTCGTTGTTCTCGG
