# Supplementary figures and images for: Sox5 is involved in germ-cell regulation and sex determination in medaka following co-option of nested transposable elements
Source: BMC Biol. 2018 Jan 29;16:16. doi: 10.1186/s12915-018-0485-8 (PMC5789577; doi:10.1186/s12915-018-0485-8)

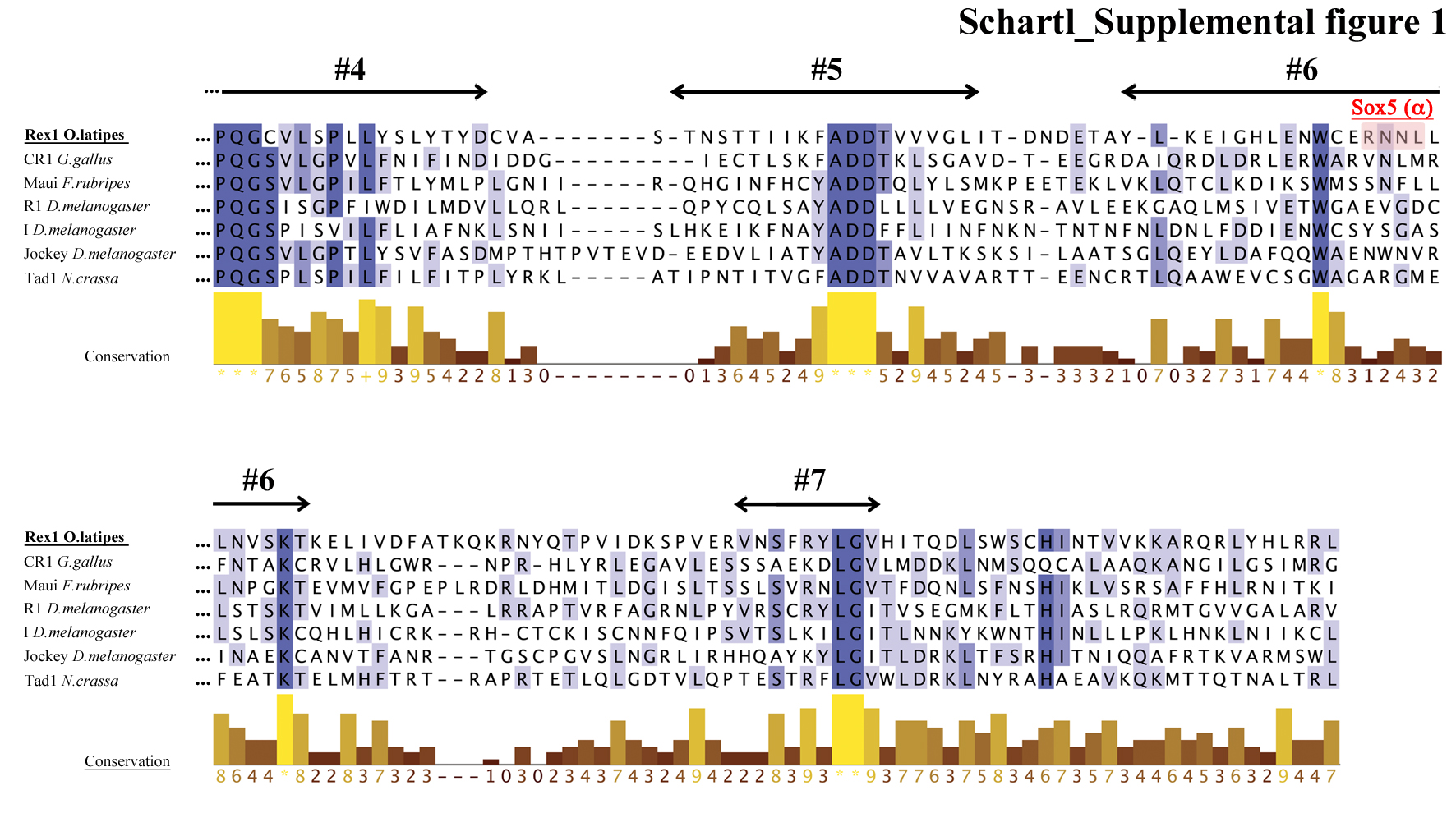

Supplement: Supplementary file 2 — Comparison of medaka Rex1 reverse-transcriptase (RT) sequence with other non-LTR retrotransposons. RT conserved domains are given according to Malik, Burke, and Eickbush [72]. RT sequences are CR1 from Gallus gallus (U88211); Maui from Fugu rubripes (AF086712); Jockey, R1, and I from Drosophila melanogaster (P21328, X51968, and M14954, respectively); and Tad1 from Neurospora crassa (L25662). The degree of amino acid conservation between sequences is shown at the foot of the alignment. (JPG 811 kb) [file 12915_2018_485_MOESM2_ESM.jpg]

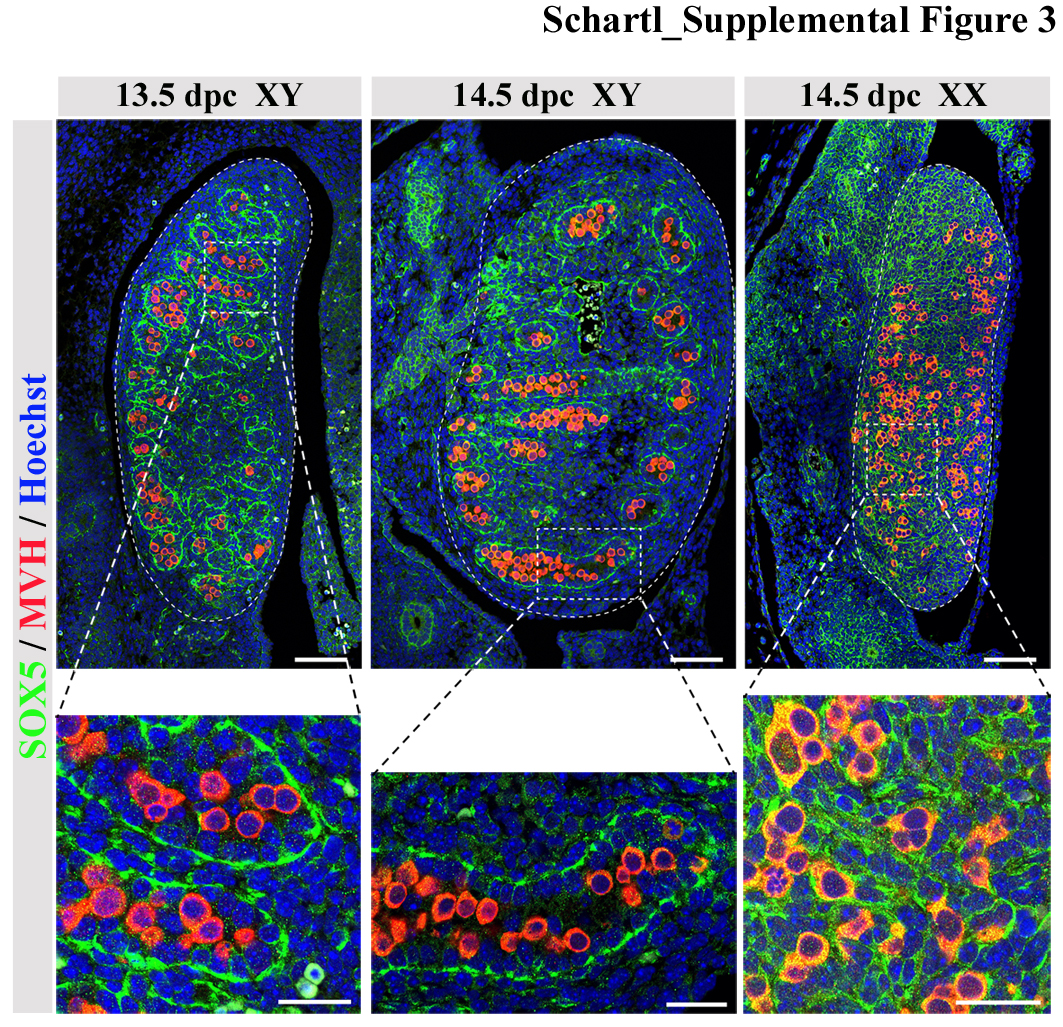

Supplement: Supplementary file 4 — SOX5 protein expression in fetal mouse gonads. Double immunofluorescence of SOX5 (green) and MVH (red) on sagittal sections of 13.5 dpc (left panel) and 14.5 dpc (middle panel) mouse testes, as well as 14.5 dpc mouse ovaries (right panel). Gonads are demarcated with dotted lines. The lower panels are a higher magnification image of the area marked by a square in the upper panels. Scale bars 100 μm (upper panels) and 30 μm (lower panels). (JPG 1710 kb) [file 12915_2018_485_MOESM4_ESM.jpg]
