# Supplementary material for: Sox5 is involved in germ-cell regulation and sex determination in medaka following co-option of nested transposable elements
Source: BMC Biol. 2018 Jan 29;16:16. doi: 10.1186/s12915-018-0485-8 (PMC5789577; doi:10.1186/s12915-018-0485-8)
Supplement: Supplementary file 3 — Location and adjacent genes of Rex1 elements containing Sox5 binding sites in the medaka genome. (PDF 70 kb) [file 12915_2018_485_MOESM3_ESM.pdf]

Supplementary Table 1: Location and adjacent genes of Rex1 elements containing Sox5 binding site in the medaka genome.

Chr.	Scaffold	Contig	within gene…		5' gene		3' gene	
?	673	104146	-	-	ENSORLT00000023673	mitochondrial sodium/hydrogen exchanger 9B2	ENSORLG00000019007	atrial natriuretic peptide-converting enzyme
12	401	93285	-	-	ENSORLG00000012002	cystatin-F	ENSORLG00000012038	ras association domain-containing protein 6
?	709	105042	-	-	-	-	-	-
18	288	83261	ENSORLG00000003201	GRB2-associated-binding protein 1	ENSORLG00000003207	round spermatid basic protein 1	-	-
3	104	49572			ENSORLG00000000988	leucine-rich repeat-containing protein 4C	ENSORLG00000001004	hydroxysteroid (17-beta) dehydrogenase 12b
7	12	11232	-	-	ENSORLG00000015772	Plexin A2	ENSORLG00000015821	plexin A2
13	11	10393	-	-	ENSORLG00000001433	G protein-coupled receptor 4	ENSORLG00000001463	microtubule associated protein
?	508	98838	ENSORLG00000018950	reverse transcriptase	-	-	ENSORLG00000018981	reverse transcriptase
24	301	84755	-	-	ENSORLG00000017683	mannosyl-oligosaccharide 1,2-alpha-mannosidase IA	-	-
11	92	46375	ENSORLG00000002385	solute carrier family 4				
16	391	92555	ENSORLG00000016896	semaphoring 4Aa	-	-	-	-
?	628	102914	-	-			ENSORLG00000018864	myelin
8	42	28227	-	-	ENSORLG00000014613	potassium voltage-gated channel subfamily J member 4	ENSORLG00000014623	endoplasmic reticulum protein
?	616	102577	ENSORLG00000019210	Unkknown				
5	367	90760	-	-	ENSORLG00000002773	transposon TX1 protein	ENSORLG00000002821	sphingomyelin phosphodiesterase 4
?	2071		-	-	-	-	-	-
?	724	105367	-	-	-	-	-	-
23	410	93862	-	-	ENSORLG00000012160	nucleosome assembly protein 1	ENSORLG00000012195	oxysterol binding protein 8
